# Supplementary material for: Molecular Adaptations to Repeated Radiation Exposure in Triple-Negative Breast Cancer: Dysregulation of Cell Adhesion, Mitochondrial Function, and Epithelial–Mesenchymal Transition
Source: Int J Mol Sci. 2025 Oct 1;26(19):9611. doi: 10.3390/ijms26199611 (PMC12525276; doi:10.3390/ijms26199611)
Supplement: Supplementary file 1 [file ijms-26-09611-s001.zip › ijms-3883560-supplementary.pdf]

Supplementary Tables

Supplementary Table S1. Differentially expressed genes in MDA-MB-231^{RR} cells relative to MDA-MB-231^{RR} cells. Genes were considered differentially expressed if they exhibited a fold change (F.C.) >2 or <–2, with an FDR-adjusted p-value (p-adj) < 0.05 and a minimum average transcript abundance of 40 transcripts per million (TPM). This table includes gene names, fold changes, p-adj values, and transcript abundance (TPM). A total of 1,572 DEGs were identified, consisting of 547 upregulated and 1,025 downregulated genes. Genes are ranked by fold change.

Gene ID	Gene Name	F.C.	p-adj.	Control Cells	RR Cells
ENSG00000129682.16	*FGF13*	3914.8	1.15E-44	0.17	684.36
ENSG00000136352.17	*NKX2-1*	2311.0	1.87E-39	0.17	403.98
ENSG00000082397.17	*EPB41L3*	889.9	4.38E-19	0.42	370.63
ENSG00000229415.9	*SFTA3*	748.6	2.62E-28	0.17	130.83
ENSG00000184515.11	*BEX5*	546.8	3.37E-26	0.24	133.82
ENSG00000154330.13	*PGM5*	451.4	3.21E-48	1.60	723.15
ENSG00000183580.10	*FBXL7*	300.6	1.25E-20	0.25	74.68
ENSG00000144868.13	*TMEM108*	263.1	6.47E-19	0.24	63.97
ENSG00000147206.17	*NXF3*	262.1	1.71E-26	0.60	156.14
ENSG00000140379.8	*BCL2A1*	237.1	3.99E-18	0.17	41.30
ENSG00000147246.10	*HTR2C*	230.7	6.43E-19	0.17	40.25
ENSG00000151892.14	*GFRA1*	187.3	1.57E-28	0.79	148.66
ENSG00000186462.9	*NAP1L2*	146.8	1.53E-15	0.47	69.22
ENSG00000163376.11	*KBTBD8*	112.0	4.55E-14	0.44	49.39
ENSG00000168952.15	*STXBP6*	108.6	8.45E-162	4.74	515.09
ENSG00000149968.12	*MMP3*	99.8	9.69E-31	1.80	179.12
ENSG00000165025.15	*SYK*	98.5	8.66E-16	0.55	54.15
ENSG00000143333.7	*RGS16*	70.1	4.77E-15	0.63	44.14
ENSG00000163017.14	*ACTG2*	51.0	2.65E-18	0.93	47.58
ENSG00000212725.3	*KRTAP2-1*	50.0	1.94E-32	2.53	126.57
ENSG00000110002.16	*VWA5A*	48.2	1.72E-62	3.70	178.23
ENSG00000044524.11	*EPHA3*	47.7	5.49E-32	2.42	115.28
ENSG00000105851.11	*PIK3CG*	45.4	7.19E-57	3.41	155.06
ENSG00000055813.6	*CCDC85A*	44.9	6.96E-26	1.42	64.00
ENSG00000038427.16	*VCAN*	43.6	2.85E-30	5.90	257.42
ENSG00000196611.5	*MMP1*	42.5	7.82E-71	167.77	7136.28
ENSG00000212724.3	*KRTAP2-3*	39.6	1.99E-57	32.21	1275.33
ENSG00000203930.12	*LINC00632*	37.5	6.92E-37	6.65	249.43
ENSG00000213417.3	*KRTAP2-4*	33.7	1.65E-22	2.07	69.70
ENSG00000203926.5	*SPANXA2*	33.7	6.10E-58	8.06	271.18
ENSG00000165323.15	*FAT3*	33.6	9.32E-38	25.48	855.06
ENSG00000136167.14	*LCP1*	30.1	1.16E-104	43.35	1304.46
ENSG00000198626.17	*RYR2*	29.2	2.86E-24	2.41	70.33
ENSG00000165105.10	*RASEF*	28.2	1.64E-67	5.62	158.29
ENSG00000198157.11	*HMGN5*	25.5	5.53E-241	27.18	692.53

ENSG00000154917.11	*RAB6B*	23.6	2.84E-16	5.09	120.26
ENSG00000115616.3	*SLC9A2*	21.0	3.07E-31	3.48	73.12
ENSG00000123572.17	*NRK*	19.2	3.80E-240	103.03	1980.08
ENSG00000073756.12	*PTGS2*	16.7	2.32E-06	17.95	300.21
ENSG00000198203.10	*SULT1C2*	16.6	6.90E-11	2.99	49.65
ENSG00000104490.18	*NCALD*	16.5	8.57E-69	10.13	167.28
ENSG00000146122.17	*DAAM2*	16.4	2.15E-32	14.97	245.30
ENSG00000165259.14	*HDX*	16.3	2.88E-37	8.63	141.16
ENSG00000184156.17	*KCNQ3*	16.3	3.60E-91	20.96	341.85
ENSG00000107282.8	*APBA1*	16.1	3.73E-07	2.95	47.49
ENSG00000170439.7	*METTL7B*	15.7	7.01E-19	2.70	42.46
ENSG00000102349.18	*KLF8*	15.6	7.25E-88	13.73	214.81
ENSG00000101680.15	*LAMA1*	15.4	7.24E-26	8.74	134.37
ENSG00000176658.17	*MYO1D*	15.0	2.59E-17	9.89	148.33
ENSG00000215374.6	*FAM66B*	13.5	5.42E-33	8.07	108.85
ENSG00000154678.18	*PDE1C*	13.3	1.28E-46	17.09	227.86
ENSG00000069667.16	*RORA*	13.3	1.96E-13	6.08	80.63
ENSG00000175928.6	*LRRN1*	13.2	7.83E-76	22.15	292.47
ENSG00000166394.15	*CYB5R2*	13.0	7.86E-41	7.29	95.11
ENSG00000166415.15	*WDR72*	12.1	3.41E-21	5.49	66.39
ENSG00000187243.16	*MAGED4B*	11.8	5.13E-26	24.68	291.43
ENSG00000099139.13	*PCSK5*	11.5	2.26E-20	5.76	65.99
ENSG00000187601.5	*MAGEH1*	10.4	2.03E-90	30.35	316.25
ENSG00000102359.7	*SRPX2*	10.4	4.16E-37	30.32	313.80
ENSG00000067141.17	*NEO1*	10.2	9.62E-47	78.39	797.10
ENSG00000272398.6	*CD24*	10.1	6.99E-99	203.17	2058.08
ENSG00000081051.8	*AFP*	10.1	4.51E-24	5.27	53.12
ENSG00000147036.11	*LANCL3*	9.6	6.44E-06	5.56	53.58
ENSG00000117069.15	*ST6GALNAC5*	9.4	2.48E-20	15.81	148.27
ENSG00000055163.20	*CYFIP2*	9.4	5.15E-58	28.37	265.80
ENSG00000074211.14	*PPP2R2C*	9.0	4.19E-117	36.71	332.17
ENSG00000213468.7	*FIRRE*	8.6	2.13E-31	10.09	87.10
ENSG00000117152.13	*RGS4*	8.2	6.85E-41	34.72	285.69
ENSG00000112902.12	*SEMA5A*	8.0	6.47E-14	8.33	66.96
ENSG00000116701.14	*NCF2*	7.8	2.09E-62	34.37	269.77
ENSG00000163909.8	*HEYL*	7.8	1.61E-16	5.21	40.41
ENSG00000259495.3	*AC016705.2*	7.7	2.11E-14	6.25	48.32
ENSG00000156345.17	*CDK20*	7.4	2.66E-21	10.66	79.15
ENSG00000149573.9	*MPZL2*	7.3	5.89E-12	10.38	75.80
ENSG00000170381.14	*SEMA3E*	7.3	3.73E-23	70.69	515.38
ENSG00000238266.2	*LINC00707*	7.3	6.14E-105	32.27	234.75
ENSG00000170786.12	*SDR16C5*	7.2	1.35E-17	9.48	67.93
ENSG00000166396.13	*SERPINB7*	7.2	1.03E-52	25.75	184.40
ENSG00000254979.5	*AP000781.2*	7.0	4.11E-20	7.35	51.39

ENSG00000182195.9	*LDOC1*	6.9	0.00E+00	142.11	985.07
ENSG00000165359.15	*INTS6L*	6.9	1.51E-08	31.33	215.03
ENSG00000184486.10	*POU3F2*	6.7	2.35E-53	37.23	250.60
ENSG00000115414.19	*FN1*	6.7	8.18E-155	5739.33	38178.95
ENSG00000175745.14	*NR2F1*	6.6	1.30E-136	197.01	1295.50
ENSG00000270977.2	*AC015849.5*	6.5	3.67E-09	6.71	43.48
ENSG00000184838.15	*PRR16*	6.4	2.28E-121	66.49	428.10
ENSG00000120149.9	*MSX2*	6.4	1.66E-24	8.68	55.63
ENSG00000165929.13	*TC2N*	6.4	6.79E-25	15.51	98.49
ENSG00000152377.14	*SPOCK1*	6.2	1.03E-12	23.04	143.68
ENSG00000151062.15	*CACNA2D4*	6.1	7.70E-78	37.70	231.83
ENSG00000131171.13	*SH3BGRL*	6.1	0.00E+00	358.93	2195.37
ENSG00000184867.14	*ARMCX2*	6.0	7.99E-78	282.56	1705.30
ENSG00000267405.1	*AC005180.1*	5.9	8.98E-41	17.86	105.27
ENSG00000227234.2	*SPANXB1*	5.9	1.43E-153	344.64	2030.89
ENSG00000139970.17	*RTN1*	5.8	1.27E-37	15.49	90.24
ENSG00000185860.13	*CCDC190*	5.8	5.32E-113	67.33	387.29
ENSG00000164220.7	*F2RL2*	5.7	7.22E-28	12.73	73.12
ENSG00000148926.10	*ADM*	5.6	5.12E-176	849.58	4798.21
ENSG00000255495.1	*AC145124.1*	5.6	6.35E-40	28.99	163.11
ENSG00000140545.15	*MFGE8*	5.6	3.50E-69	1368.17	7656.23
ENSG00000196335.13	*STK31*	5.3	4.58E-24	15.35	82.03
ENSG00000237943.7	*PRKCQ-AS1*	5.3	7.65E-13	14.39	76.68
ENSG00000215808.4	*LINC01139*	5.3	1.33E-44	25.63	135.91
ENSG00000139973.16	*SYT16*	5.2	2.55E-05	15.54	81.18
ENSG00000166432.15	*ZMAT1*	5.1	1.78E-12	14.60	74.39
ENSG00000160588.10	*MPZL3*	5.1	4.68E-28	25.06	127.44
ENSG00000140937.14	*CDH11*	4.9	3.40E-59	656.02	3219.48
ENSG00000140450.9	*ARRDC4*	4.9	1.71E-36	55.70	272.29
ENSG00000139946.10	*PELI2*	4.8	1.68E-37	23.95	116.12
ENSG00000105409.19	*ATP1A3*	4.8	3.66E-65	103.78	502.86
ENSG00000036448.10	*MYOM2*	4.8	1.67E-30	18.19	87.63
ENSG00000198934.5	*MAGEE1*	4.8	5.59E-73	53.91	259.51
ENSG00000063169.10	*BICRA*	4.8	4.65E-06	12.22	58.72
ENSG00000135828.11	*RNASEL*	4.7	5.58E-23	15.18	71.91
ENSG00000129595.13	*EPB41L4A*	4.7	2.50E-25	34.13	160.14
ENSG00000251281.1	*AC034223.2*	4.6	1.71E-17	11.76	54.40
ENSG00000120129.6	*DUSP1*	4.6	1.17E-112	558.73	2558.69
ENSG00000117143.13	*UAP1*	4.6	1.22E-09	17.18	78.52
ENSG00000110693.18	*SOX6*	4.5	1.38E-08	10.74	48.75
ENSG00000168497.5	*CAVIN2*	4.5	7.81E-28	941.21	4249.12
ENSG00000186517.14	*ARHGAP30*	4.5	1.31E-16	11.95	53.90
ENSG00000203721.7	*LINC00862*	4.5	2.11E-11	9.39	42.27
ENSG00000182621.18	*PLCB1*	4.4	2.84E-26	77.85	341.39

ENSG00000132639.12	*SNAP25*	4.4	2.44E-07	12.57	54.93
ENSG00000187957.8	*DNER*	4.4	9.04E-34	37.25	162.41
ENSG00000087258.15	*GNAO1*	4.3	8.97E-39	74.36	322.88
ENSG00000057657.17	*PRDM1*	4.3	5.14E-14	13.29	57.60
ENSG00000281091.3	*AL117327.1*	4.3	1.19E-11	10.76	45.71
ENSG00000118503.15	*TNFAIP3*	4.2	1.78E-23	96.87	411.35
ENSG00000156959.9	*LHFPL4*	4.2	1.27E-13	43.23	182.22
ENSG00000166387.13	*PPFIBP2*	4.2	1.50E-11	17.50	73.74
ENSG00000153237.18	*CCDC148*	4.2	5.88E-07	9.91	41.54
ENSG00000129675.16	*ARHGEF6*	4.2	3.77E-13	13.92	57.82
ENSG00000135363.12	*LMO2*	4.1	1.05E-13	10.45	43.31
ENSG00000258659.6	*TRIM34*	4.1	3.48E-32	130.60	539.80
ENSG00000283486.2	*FAM95C*	4.1	3.46E-05	14.78	61.06
ENSG00000213694.5	*S1PR3*	4.1	4.30E-75	125.06	515.60
ENSG00000183496.6	*MEX3B*	4.1	2.66E-25	39.32	162.05
ENSG00000258818.4	*RNASE4*	4.1	4.44E-03	10.00	41.09
ENSG00000172201.12	*ID4*	4.1	1.08E-20	58.45	239.42
ENSG00000198963.11	*RORB*	4.1	7.47E-20	18.96	77.06
ENSG00000184613.10	*NELL2*	4.1	4.89E-05	14.77	59.86
ENSG00000144834.14	*TAGLN3*	4.0	4.58E-13	11.49	46.33
ENSG00000198932.13	*GPRASP1*	4.0	2.80E-16	12.44	50.02
ENSG00000198960.11	*ARMCX6*	4.0	1.68E-199	465.58	1867.97
ENSG00000163412.13	*EIF4E3*	4.0	2.83E-36	40.47	161.82
ENSG00000177839.7	*PCDHB9*	4.0	2.77E-05	15.34	61.11
ENSG00000106733.21	*NMRK1*	4.0	7.95E-34	37.35	147.76
ENSG00000018610.14	*CXorf56*	3.9	4.90E-11	46.24	181.78
ENSG00000162981.14	*LRATD1*	3.9	2.35E-15	21.37	84.01
ENSG00000179222.17	*MAGED1*	3.9	3.60E-37	142.97	560.70
ENSG00000144810.16	*COL8A1*	3.9	1.52E-159	1785.15	6997.30
ENSG00000132359.15	*RAP1GAP2*	3.9	1.44E-56	195.76	762.04
ENSG00000241472.7	*PTPRG-AS1*	3.9	1.21E-18	75.40	292.55
ENSG00000134602.16	*STK26*	3.9	1.16E-189	240.89	934.06
ENSG00000173530.6	*TNFRSF10D*	3.8	6.06E-165	533.21	2052.84
ENSG00000135905.19	*DOCK10*	3.8	5.64E-03	55.26	209.30
ENSG00000169851.15	*PCDH7*	3.8	4.17E-95	196.33	742.45
ENSG00000168685.15	*IL7R*	3.8	3.95E-63	245.19	926.84
ENSG00000147231.14	*RADX*	3.8	1.29E-22	63.93	239.90
ENSG00000102024.18	*PLS3*	3.7	0.00E+00	4208.99	15708.57
ENSG00000116183.11	*PAPPA2*	3.7	4.48E-16	25.13	93.40
ENSG00000172159.16	*FRMD3*	3.7	2.28E-33	69.22	257.13
ENSG00000206538.9	*VGLL3*	3.7	9.64E-19	51.05	188.69
ENSG00000133138.20	*TBC1D8B*	3.7	7.21E-26	74.14	271.19
ENSG00000041982.16	*TNC*	3.6	5.82E-62	740.72	2698.13
ENSG00000180964.17	*TCEAL8*	3.6	1.79E-95	194.68	704.78

ENSG00000069869.16	*NEDD4*	3.6	1.09E-16	232.65	838.48
ENSG00000012124.17	*CD22*	3.6	1.14E-40	121.08	432.79
ENSG00000189056.14	*RELN*	3.6	1.43E-05	27.57	98.28
ENSG00000103528.17	*SYT17*	3.5	9.04E-13	32.64	115.72
ENSG00000143473.13	*KCNH1*	3.5	3.65E-08	15.10	53.33
ENSG00000257219.6	*LNCOG*	3.5	2.41E-11	11.65	41.07
ENSG00000225383.8	*SFTA1P*	3.5	1.00E-51	264.47	923.37
ENSG00000154734.15	*ADAMTS1*	3.5	7.86E-42	553.84	1929.87
ENSG00000135750.15	*KCNK1*	3.5	2.71E-54	127.19	443.12
ENSG00000182983.15	*ZNF662*	3.5	8.21E-18	21.05	73.26
ENSG00000251273.4	*LINC02228*	3.5	1.61E-33	43.04	149.76
ENSG00000269226.7	*TMSB15B*	3.5	5.86E-23	29.44	101.67
ENSG00000197632.9	*SERPINB2*	3.4	5.40E-14	16.48	56.67
ENSG00000101974.14	*ATP11C*	3.4	3.37E-15	207.95	713.98
ENSG00000271147.8	*ARMCX5-GPRASP2*	3.4	1.58E-18	35.21	120.50
ENSG00000147202.18	*DIAPH2*	3.4	1.16E-113	216.83	738.45
ENSG00000250697.2	*AC010343.3*	3.4	2.83E-49	129.19	434.66
ENSG00000109861.16	*CTSC*	3.3	0.00E+00	2232.24	7457.90
ENSG00000147251.15	*DOCK11*	3.3	2.45E-06	55.32	184.46
ENSG00000223749.10	*MIR503HG*	3.3	9.36E-25	57.63	191.27
ENSG00000173110.8	*HSPA6*	3.3	2.37E-11	18.42	61.00
ENSG00000094841.14	*UPRT*	3.3	3.68E-53	135.15	446.24
ENSG00000187554.14	*TLR5*	3.3	8.48E-04	12.69	41.52
ENSG00000170271.11	*FAXDC2*	3.3	5.35E-06	13.84	45.24
ENSG00000235904.3	*RBMS3-AS3*	3.3	1.39E-25	35.41	115.58
ENSG00000147100.11	*SLC16A2*	3.3	4.34E-15	26.13	84.97
ENSG00000165240.20	*ATP7A*	3.2	2.58E-06	46.09	148.62
ENSG00000115363.14	*EVA1A*	3.2	1.81E-74	339.78	1089.77
ENSG00000156463.18	*SH3RF2*	3.2	5.91E-93	562.65	1792.71
ENSG00000143502.15	*SUSD4*	3.2	9.00E-06	19.21	61.18
ENSG00000149591.17	*TAGLN*	3.2	1.63E-25	289.26	916.84
ENSG00000102390.11	*PBDC1*	3.2	1.80E-105	591.86	1872.74
ENSG00000149212.12	*SESN3*	3.2	5.74E-32	95.52	302.21
ENSG00000236824.2	*BCYRN1*	3.1	1.00E-31	1538.81	4842.67
ENSG00000079841.18	*RIMS1*	3.1	1.49E-06	20.15	63.24
ENSG00000125352.6	*RNF113A*	3.1	3.59E-41	244.41	765.90
ENSG00000189369.9	*GSPT2*	3.1	2.91E-66	116.97	364.78
ENSG00000168386.18	*FILIP1L*	3.1	1.29E-46	112.44	349.88
ENSG00000230615.7	*AL139220.2*	3.1	3.70E-08	21.04	65.29
ENSG00000257529.5	*RPL36A-HNRNPH2*	3.1	3.77E-14	188.47	583.37
ENSG00000117525.14	*F3*	3.1	1.11E-193	7230.71	22303.60
ENSG00000139971.15	*ARMH4*	3.1	9.30E-21	77.31	236.04

ENSG00000166833.21	*NAV2*	3.0	6.23E-07	551.42	1677.15
ENSG00000166352.16	*C11orf74*	3.0	4.04E-07	51.18	155.36
ENSG00000126947.13	*ARMCX1*	3.0	6.37E-92	331.62	1003.25
ENSG00000167772.12	*ANGPTL4*	3.0	3.70E-74	125.04	375.99
ENSG00000169116.11	*PARM1*	3.0	4.76E-20	68.99	207.29
ENSG00000272870.3	*AC097534.2*	3.0	2.08E-04	17.37	52.17
ENSG00000240747.7	*KRBOX1*	3.0	5.92E-03	17.17	51.56
ENSG00000171033.13	*PKIA*	3.0	1.17E-61	215.80	647.62
ENSG00000259171.1	*AL163636.2*	3.0	3.93E-06	22.81	68.23
ENSG00000143429.10	*LSP1P4*	3.0	2.97E-03	15.58	46.58
ENSG00000228340.6	*MIR646HG*	3.0	5.37E-39	66.51	196.61
ENSG00000154589.7	*LY96*	3.0	2.04E-06	17.54	51.79
ENSG00000170961.7	*HAS2*	2.9	7.02E-56	449.57	1321.95
ENSG00000188706.13	*ZDHHC9*	2.9	3.11E-214	614.67	1805.35
ENSG00000140416.21	*TPM1*	2.9	0.00E+00	1072.08	3143.22
ENSG00000134668.12	*SPOCD1*	2.9	5.96E-34	131.02	383.43
ENSG00000170006.12	*TMEM154*	2.9	2.08E-09	100.32	293.26
ENSG00000230043.1	*TMSB4XP6*	2.9	4.18E-06	17.38	50.78
ENSG00000162733.19	*DDR2*	2.9	5.28E-08	22.16	64.55
ENSG00000184164.14	*CRELD2*	2.9	7.33E-07	20.07	58.17
ENSG00000132406.12	*TMEM128*	2.9	1.36E-85	215.88	623.15
ENSG00000134597.16	*RBMX2*	2.9	3.30E-151	332.31	954.47
ENSG00000277701.5	*AC159540.2*	2.9	9.74E-06	15.21	43.65
ENSG00000069974.16	*RAB27A*	2.9	8.61E-81	383.26	1099.54
ENSG00000101856.10	*PGRMC1*	2.9	1.45E-222	1183.04	3390.03
ENSG00000269918.1	*AF131215.6*	2.9	8.89E-11	17.79	50.87
ENSG00000203950.6	*RTL8A*	2.9	1.90E-14	115.19	329.11
ENSG00000026751.17	*SLAMF7*	2.8	4.02E-70	539.81	1531.46
ENSG00000177485.7	*ZBTB33*	2.8	4.49E-147	372.60	1056.89
ENSG00000183287.14	*CCBE1*	2.8	4.95E-66	386.77	1096.92
ENSG00000154889.17	*MPPE1*	2.8	6.85E-13	30.94	87.72
ENSG00000145358.6	*DDIT4L*	2.8	1.05E-07	18.48	52.38
ENSG00000181751.10	*C5orf30*	2.8	2.98E-173	397.58	1125.82
ENSG00000120324.9	*PCDHB10*	2.8	2.82E-09	18.73	52.82
ENSG00000231527.7	*FAM27C*	2.8	4.26E-18	45.89	129.17
ENSG00000144642.22	*RBMS3*	2.8	5.67E-48	225.45	632.76
ENSG00000170498.9	*KISS1*	2.8	2.86E-16	304.13	853.06
ENSG00000235174.1	*RPL39P3*	2.8	1.50E-12	28.30	79.21
ENSG00000138675.16	*FGF5*	2.8	2.40E-10	330.37	923.36
ENSG00000175556.17	*LONRF3*	2.8	2.06E-44	135.68	378.62
ENSG00000118508.5	*RAB32*	2.8	8.66E-136	717.02	1994.76
ENSG00000198908.12	*BHLHB9*	2.8	2.03E-31	99.17	274.81
ENSG00000067606.17	*PRKCZ*	2.8	3.46E-39	147.64	408.97
ENSG00000165675.16	*ENOX2*	2.8	2.33E-08	45.05	124.71

ENSG00000171522.6	*PTGER4*	2.8	3.03E-70	176.60	488.70
ENSG00000188419.14	*CHM*	2.8	6.24E-133	381.27	1051.22
ENSG00000232119.8	*MCTS1*	2.7	1.53E-79	727.81	1993.78
ENSG00000077713.19	*SLC25A43*	2.7	3.55E-87	393.12	1076.18
ENSG00000102401.20	*ARMCX3*	2.7	1.78E-131	398.40	1086.35
ENSG00000277734.8	*TRAC*	2.7	8.37E-12	22.02	60.01
ENSG00000115598.10	*IL1RL2*	2.7	1.82E-11	19.15	51.93
ENSG00000110031.12	*LPXN*	2.7	2.94E-04	43.94	119.10
ENSG00000185650.9	*ZFP36L1*	2.7	4.82E-07	45.50	123.18
ENSG00000214274.9	*ANG*	2.7	2.81E-09	17.62	47.67
ENSG00000140009.18	*ESR2*	2.7	2.12E-08	14.98	40.51
ENSG00000102078.16	*SLC25A14*	2.7	1.37E-30	86.57	233.71
ENSG00000080493.17	*SLC4A4*	2.7	7.93E-13	73.00	195.72
ENSG00000134242.16	*PTPN22*	2.7	1.85E-13	26.76	71.63
ENSG00000168032.10	*ENTPD3*	2.7	4.75E-36	72.95	195.12
ENSG00000196954.14	*CASP4*	2.7	1.17E-106	518.15	1385.37
ENSG00000114405.10	*C3orf14*	2.7	4.17E-101	295.40	786.89
ENSG00000151623.15	*NR3C2*	2.7	5.68E-31	115.45	307.34
ENSG00000132109.10	*TRIM21*	2.7	5.62E-64	240.69	640.06
ENSG00000129680.16	*MAP7D3*	2.7	1.02E-86	1224.15	3254.22
ENSG00000167552.14	*TUBA1A*	2.7	2.46E-38	964.71	2562.48
ENSG00000105404.11	*RABAC1*	2.7	2.73E-184	635.72	1687.80
ENSG00000049759.18	*NEDD4L*	2.6	1.62E-103	2629.25	6965.25
ENSG00000134590.14	*RTL8C*	2.6	8.31E-143	882.54	2335.95
ENSG00000277778.2	*PGM5P2*	2.6	4.22E-08	54.08	142.98
ENSG00000131724.11	*IL13RA1*	2.6	8.54E-83	1142.41	3017.11
ENSG00000075711.21	*DLG1*	2.6	2.37E-168	2408.62	6357.22
ENSG00000197261.11	*C6orf141*	2.6	2.43E-12	48.95	129.03
ENSG00000123562.17	*MORF4L2*	2.6	4.35E-203	3483.20	9151.38
ENSG00000113578.18	*FGF1*	2.6	4.33E-10	107.92	283.05
ENSG00000050438.17	*SLC4A8*	2.6	9.41E-09	62.06	162.73
ENSG00000156697.13	*UTP14A*	2.6	1.54E-202	814.10	2131.58
ENSG00000113494.17	*PRLR*	2.6	1.94E-07	42.86	112.18
ENSG00000175390.14	*EIF3F*	2.6	1.71E-26	155.54	405.78
ENSG00000198689.11	*SLC9A6*	2.6	2.18E-06	45.42	118.48
ENSG00000171346.16	*KRT15*	2.6	2.41E-10	50.55	131.74
ENSG00000068615.18	*REEP1*	2.6	1.11E-08	27.96	72.81
ENSG00000064692.19	*SNCAIP*	2.6	3.12E-14	92.93	241.72
ENSG00000104419.14	*NDRG1*	2.6	1.53E-04	47.54	123.45
ENSG00000131773.14	*KHDRBS3*	2.6	4.27E-162	738.49	1915.61
ENSG00000077721.16	*UBE2A*	2.6	1.22E-240	1481.97	3842.43
ENSG00000076770.14	*MBNL3*	2.6	2.82E-42	169.07	437.26
ENSG00000241489.8	*AC244197.3*	2.6	2.07E-03	28.94	74.70
ENSG00000164181.14	*ELOVL7*	2.6	6.65E-16	101.80	262.60

ENSG00000186376.15	*ZNF75D*	2.6	2.79E-39	122.91	316.82
ENSG00000131018.24	*SYNE1*	2.6	3.99E-14	545.41	1404.41
ENSG00000204019.5	*CT83*	2.6	1.17E-18	49.05	126.27
ENSG00000213853.10	*EMP2*	2.6	2.64E-115	791.42	2036.21
ENSG00000135477.11	*KRT87P*	2.6	5.84E-13	54.08	139.05
ENSG00000197442.10	*MAP3K5*	2.6	4.60E-11	137.49	351.95
ENSG00000181982.18	*CCDC149*	2.6	4.30E-18	138.27	353.79
ENSG00000187534.6	*PRR13P5*	2.6	2.66E-05	19.82	50.55
ENSG00000204758.8	*AC008429.1*	2.5	1.08E-05	17.20	43.73
ENSG00000162407.9	*PLPP3*	2.5	5.70E-54	513.03	1302.86
ENSG00000135549.15	*PKIB*	2.5	6.03E-11	62.69	158.63
ENSG00000131263.13	*RLIM*	2.5	4.60E-71	782.51	1977.94
ENSG00000198478.8	*SH3BGRL2*	2.5	7.87E-19	66.09	166.88
ENSG00000186854.11	*TRABD2A*	2.5	2.40E-31	156.47	394.86
ENSG00000155008.14	*APOOL*	2.5	2.16E-99	307.52	776.00
ENSG00000125356.7	*NDUFA1*	2.5	3.87E-131	808.42	2035.52
ENSG00000227295.2	*ELL2P1*	2.5	2.62E-09	16.51	41.49
ENSG00000241343.9	*RPL36A*	2.5	8.22E-75	3043.20	7645.36
ENSG00000284948.1	*AC107959.4*	2.5	7.86E-08	18.51	46.42
ENSG00000173275.13	*ZNF449*	2.5	3.30E-16	69.57	174.47
ENSG00000230316.7	*FEZF1-AS1*	2.5	3.39E-05	29.82	74.68
ENSG00000162614.18	*NEXN*	2.5	4.52E-50	680.98	1704.86
ENSG00000245146.7	*MALINC1*	2.5	2.85E-06	24.08	60.16
ENSG00000181458.10	*TMEM45A*	2.5	7.13E-30	90.43	225.83
ENSG00000247151.7	*CSTF3-DT*	2.5	3.94E-06	17.37	43.29
ENSG00000168077.14	*SCARA3*	2.5	1.04E-22	428.52	1066.29
ENSG00000225265.2	*TAF1A-AS1*	2.5	1.33E-04	17.10	42.47
ENSG00000106034.18	*CPED1*	2.5	1.97E-36	328.20	814.28
ENSG00000197415.12	*VEPH1*	2.5	4.64E-28	122.26	303.24
ENSG00000069020.18	*MAST4*	2.5	1.08E-08	94.27	233.77
ENSG00000163568.15	*AIM2*	2.5	3.64E-15	41.84	103.64
ENSG00000205542.11	*TMSB4X*	2.5	3.03E-193	27009.58	66819.87
ENSG00000173890.17	*GPR160*	2.5	5.46E-10	62.75	155.22
ENSG00000186523.14	*FAM86B1*	2.5	7.11E-06	44.75	110.69
ENSG00000125962.14	*ARMCX5*	2.5	4.59E-04	39.76	98.31
ENSG00000120306.11	*CYSTM1*	2.5	1.46E-36	261.74	645.85
ENSG00000237187.9	*NR2F1-AS1*	2.5	6.54E-44	200.29	494.21
ENSG00000068366.20	*ACSL4*	2.5	7.02E-232	2357.47	5816.00
ENSG00000135299.17	*ANKRD6*	2.5	2.33E-18	55.56	136.87
ENSG00000255310.2	*AF131215.5*	2.5	4.41E-12	34.30	84.45
ENSG00000116133.13	*DHCR24*	2.5	4.45E-61	4437.28	10923.36
ENSG00000151611.16	*MMAA*	2.5	7.71E-04	18.52	45.50
ENSG00000205181.7	*LINC00654*	2.5	1.00E-13	79.48	194.91
ENSG00000102081.14	*FMR1*	2.4	5.18E-03	168.69	412.52

ENSG00000127252.7	*PLAAT1*	2.4	1.46E-07	25.36	61.97
ENSG00000170917.14	*NUDT6*	2.4	8.40E-23	109.39	267.12
ENSG00000160886.13	*LY6K*	2.4	4.36E-21	63.14	154.15
ENSG00000198918.8	*RPL39*	2.4	6.13E-99	4499.32	10980.62
ENSG00000170523.3	*KRT83*	2.4	1.94E-08	35.96	87.75
ENSG00000223551.1	*TMSB4XP4*	2.4	3.97E-05	17.63	43.02
ENSG00000244398.1	*AC116533.1*	2.4	3.31E-94	1901.98	4638.68
ENSG00000126953.8	*TIMM8A*	2.4	1.31E-55	343.52	836.17
ENSG00000102144.15	*PGK1*	2.4	1.40E-116	9376.44	22804.22
ENSG00000272410.5	*AC022384.1*	2.4	3.99E-06	97.75	237.57
ENSG00000120217.14	*CD274*	2.4	7.50E-140	640.56	1555.18
ENSG00000091986.15	*CCDC80*	2.4	4.73E-67	887.32	2152.56
ENSG00000177034.16	*MTX3*	2.4	9.73E-03	51.45	124.71
ENSG00000186480.13	*INSIG1*	2.4	4.27E-13	527.01	1276.70
ENSG00000143353.12	*LYPLAL1*	2.4	2.68E-14	170.04	410.80
ENSG00000231365.6	*WARS2-AS1*	2.4	3.53E-33	278.46	671.59
ENSG00000180543.5	*TSPYL5*	2.4	3.48E-28	119.03	286.34
ENSG00000100612.14	*DHRS7*	2.4	3.74E-91	380.86	915.06
ENSG00000157514.16	*TSC22D3*	2.4	3.94E-15	96.97	232.90
ENSG00000100345.21	*MYH9*	2.4	5.05E-21	208.35	500.28
ENSG00000101928.13	*MOSPD1*	2.4	2.20E-51	304.65	728.72
ENSG00000157064.11	*NMNAT2*	2.4	1.29E-23	278.77	666.32
ENSG00000146278.11	*PNRC1*	2.4	2.80E-54	203.01	485.06
ENSG00000155962.13	*CLIC2*	2.4	1.24E-03	18.13	43.28
ENSG00000254837.2	*AP001372.2*	2.4	9.43E-09	35.57	84.54
ENSG00000164619.10	*BMPER*	2.4	3.34E-07	39.29	93.02
ENSG00000165511.6	*C10orf25*	2.4	3.15E-11	33.22	78.43
ENSG00000121361.5	*KCNJ8*	2.4	1.79E-07	27.54	64.85
ENSG00000171914.16	*TLN2*	2.4	3.63E-09	210.98	496.70
ENSG00000277147.6	*LINC00869*	2.3	6.97E-08	44.29	103.95
ENSG00000114850.6	*SSR3*	2.3	9.80E-79	1276.83	2991.89
ENSG00000169299.14	*PGM2*	2.3	1.01E-213	1785.74	4183.81
ENSG00000215086.2	*NPM1P24*	2.3	1.09E-07	33.24	77.69
ENSG00000123728.10	*RAP2C*	2.3	2.03E-74	553.47	1283.76
ENSG00000212747.5	*RTL8B*	2.3	4.53E-58	355.05	823.30
ENSG00000005893.15	*LAMP2*	2.3	5.46E-127	1473.07	3413.07
ENSG00000179833.4	*SERTAD2*	2.3	3.12E-64	1445.60	3348.06
ENSG00000153944.11	*MSI2*	2.3	4.92E-05	85.98	199.03
ENSG00000166147.13	*FBN1*	2.3	1.66E-22	290.88	672.78
ENSG00000111875.8	*ASF1A*	2.3	1.38E-70	512.97	1186.08
ENSG00000133142.17	*TCEAL4*	2.3	8.80E-13	308.51	712.96
ENSG00000102241.12	*HTATSF1*	2.3	1.16E-215	1293.85	2989.24
ENSG00000223745.8	*CCDC18-AS1*	2.3	2.76E-04	31.84	73.52
ENSG00000143479.17	*DYRK3*	2.3	8.33E-33	145.32	335.21

ENSG00000187098.15	*MITF*	2.3	5.96E-34	433.99	1000.31
ENSG00000215105.4	*TTC3P1*	2.3	7.32E-08	28.64	65.99
ENSG00000080561.13	*MID2*	2.3	6.17E-07	22.25	51.17
ENSG00000113108.19	*APBB3*	2.3	9.69E-09	62.65	143.88
ENSG00000212978.6	*AC016747.1*	2.3	2.87E-14	71.75	164.62
ENSG00000188064.10	*WNT7B*	2.3	2.14E-39	832.55	1909.81
ENSG00000141655.17	*TNFRSF11A*	2.3	1.89E-40	289.86	663.80
ENSG00000101194.18	*SLC17A9*	2.3	6.69E-86	1192.49	2730.01
ENSG00000138798.13	*EGF*	2.3	2.20E-05	53.48	122.29
ENSG00000180801.14	*ARSJ*	2.3	9.11E-66	1302.60	2976.83
ENSG00000241749.4	*RPSAP52*	2.3	2.06E-12	36.82	84.14
ENSG00000102316.17	*MAGED2*	2.3	4.33E-87	1936.20	4421.40
ENSG00000143198.13	*MGST3*	2.3	2.21E-129	697.45	1591.69
ENSG00000164161.10	*HHIP*	2.3	4.63E-05	85.74	195.48
ENSG00000185222.10	*TCEAL9*	2.3	6.38E-97	892.33	2034.00
ENSG00000249673.7	*NOP14-AS1*	2.3	3.37E-42	328.59	748.28
ENSG00000184292.7	*TACSTD2*	2.3	1.18E-136	597.48	1359.10
ENSG00000160255.18	*ITGB2*	2.3	3.55E-07	118.16	268.52
ENSG00000101844.18	*ATG4A*	2.3	4.97E-31	210.12	477.10
ENSG00000180287.16	*PLD5*	2.3	9.51E-16	81.19	184.27
ENSG00000205464.12	*ATP6AP1L*	2.3	1.00E-07	27.97	63.46
ENSG00000223784.2	*LINP1*	2.3	4.34E-16	162.22	367.72
ENSG00000053501.13	*USE1*	2.3	4.48E-45	156.58	354.65
ENSG00000131174.7	*COX7B*	2.3	2.00E-66	954.79	2160.29
ENSG00000120738.8	*EGR1*	2.3	1.63E-08	39.27	88.84
ENSG00000196352.15	*CD55*	2.3	4.75E-86	2575.53	5825.26
ENSG00000164442.10	*CITED2*	2.3	4.32E-36	1732.87	3912.94
ENSG00000131269.19	*ABCB7*	2.3	8.66E-35	288.57	651.09
ENSG00000170571.12	*EMB*	2.3	8.37E-67	497.04	1120.52
ENSG00000101843.19	*PSMD10*	2.3	3.51E-41	682.05	1537.02
ENSG00000145349.17	*CAMK2D*	2.3	1.42E-13	354.92	799.32
ENSG00000109743.11	*BST1*	2.3	6.90E-06	20.10	45.26
ENSG00000000003.15	*TSPAN6*	2.3	6.74E-48	408.31	919.28
ENSG00000155189.12	*AGPAT5*	2.3	7.00E-110	1166.19	2624.23
ENSG00000099860.9	*GADD45B*	2.2	3.29E-82	742.30	1669.90
ENSG00000165704.15	*HPRT1*	2.2	8.10E-201	891.48	2004.31
ENSG00000105877.18	*DNAH11*	2.2	2.66E-06	54.60	122.60
ENSG00000148057.16	*IDNK*	2.2	3.21E-31	91.13	204.45
ENSG00000083799.17	*CYLD*	2.2	4.30E-50	402.94	902.21
ENSG00000187325.5	*TAF9B*	2.2	2.83E-61	348.78	780.78
ENSG00000251669.5	*FAM86EP*	2.2	1.02E-05	33.80	75.55
ENSG00000251593.1	*MSNP1*	2.2	2.51E-11	38.73	86.45
ENSG00000145287.10	*PLAC8*	2.2	2.46E-73	568.45	1268.91
ENSG00000242732.4	*RTL5*	2.2	1.29E-26	109.35	243.61

ENSG00000174851.16	*YIF1A*	2.2	3.06E-85	669.92	1490.68
ENSG00000272622.2	*AC010735.2*	2.2	1.41E-08	43.75	97.27
ENSG00000101901.12	*ALG13*	2.2	6.93E-63	299.00	663.90
ENSG00000128016.7	*ZFP36*	2.2	1.77E-39	152.28	337.88
ENSG00000196639.6	*HRH1*	2.2	9.96E-06	69.28	153.67
ENSG00000005022.6	*SLC25A5*	2.2	3.28E-121	6954.52	15416.29
ENSG00000254413.8	*CHKB-CPT1B*	2.2	1.72E-04	30.31	67.12
ENSG00000131725.14	*WDR44*	2.2	1.41E-44	494.84	1094.78
ENSG00000048740.18	*CELF2*	2.2	8.79E-25	457.94	1012.91
ENSG00000011638.10	*TMEM159*	2.2	7.59E-06	113.15	249.85
ENSG00000118985.16	*ELL2*	2.2	2.25E-87	1288.94	2845.10
ENSG00000147065.17	*MSN*	2.2	0.00E+00	20470.13	45170.49
ENSG00000090776.6	*EFNB1*	2.2	4.34E-30	363.03	799.16
ENSG00000062282.15	*DGAT2*	2.2	6.94E-26	164.43	361.84
ENSG00000167972.14	*ABCA3*	2.2	3.12E-18	225.38	495.28
ENSG00000254505.10	*CHMP4A*	2.2	6.94E-05	37.48	82.13
ENSG00000279821.1	*AC145098.2*	2.2	1.35E-06	21.15	46.30
ENSG00000135596.18	*MICAL1*	2.2	1.25E-19	273.37	598.28
ENSG00000144821.10	*MYH15*	2.2	1.71E-05	21.36	46.75
ENSG00000179046.8	*TRIML2*	2.2	3.87E-04	20.75	45.26
ENSG00000119599.17	*DCAF4*	2.2	1.51E-21	226.62	491.61
ENSG00000101190.13	*TCFL5*	2.2	2.07E-183	774.44	1677.43
ENSG00000069424.15	*KCNAB2*	2.2	3.89E-16	163.31	353.47
ENSG00000138395.16	*CDK15*	2.2	1.12E-03	19.99	43.24
ENSG00000274026.2	*FAM27E3*	2.2	1.03E-07	27.61	59.66
ENSG00000133069.17	*TMCC2*	2.2	1.14E-15	120.52	259.47
ENSG00000102034.16	*ELF4*	2.2	8.59E-39	617.82	1329.40
ENSG00000233184.7	*AC093157.1*	2.2	3.30E-04	25.78	55.44
ENSG00000115963.13	*RND3*	2.1	2.69E-23	815.50	1752.59
ENSG00000245571.7	*FAM111A-DT*	2.1	1.51E-06	27.20	58.39
ENSG00000174808.12	*BTC*	2.1	1.57E-04	33.65	72.11
ENSG00000101132.10	*PFDN4*	2.1	1.24E-94	811.51	1737.31
ENSG00000184117.12	*NIPSNAP1*	2.1	5.73E-46	269.68	576.12
ENSG00000184500.15	*PROS1*	2.1	6.10E-04	84.09	179.54
ENSG00000109929.10	*SC5D*	2.1	1.17E-22	501.41	1070.45
ENSG00000116717.13	*GADD45A*	2.1	1.38E-101	728.86	1555.47
ENSG00000197860.10	*SGTB*	2.1	1.06E-34	284.53	606.98
ENSG00000116260.17	*QSOX1*	2.1	9.86E-126	15114.72	32168.98
ENSG00000179242.16	*CDH4*	2.1	1.69E-44	1991.37	4222.15
ENSG00000213560.4	*AC103591.1*	2.1	2.12E-08	35.57	75.33
ENSG00000153064.12	*BANK1*	2.1	5.95E-13	95.19	201.51
ENSG00000272419.6	*LINC01145*	2.1	2.22E-09	116.80	247.18
ENSG00000145147.20	*SLIT2*	2.1	4.32E-31	738.83	1563.18
ENSG00000163393.13	*SLC22A15*	2.1	7.56E-07	81.14	171.54

ENSG00000020256.20	*ZFP64*	2.1	8.92E-40	194.51	410.73
ENSG00000282057.1	*AC092807.3*	2.1	3.15E-09	72.02	152.01
ENSG00000134996.12	*OSTF1*	2.1	4.36E-89	475.66	1003.35
ENSG00000165288.11	*BRWD3*	2.1	1.58E-22	242.91	512.00
ENSG00000225377.6	*NRSN2-AS1*	2.1	1.32E-10	53.85	113.23
ENSG00000138735.16	*PDE5A*	2.1	1.55E-03	28.98	60.93
ENSG00000136999.5	*CCN3*	2.1	2.75E-18	421.08	885.12
ENSG00000087460.25	*GNAS*	2.1	1.17E-130	3778.36	7937.27
ENSG00000122126.17	*OCRL*	2.1	2.91E-187	961.33	2018.89
ENSG00000169429.11	*CXCL8*	2.1	3.41E-17	120.95	253.75
ENSG00000132749.11	*TESMIN*	2.1	1.63E-14	88.20	184.92
ENSG00000040608.14	*RTN4R*	2.1	1.30E-05	19.46	40.78
ENSG00000182718.16	*ANXA2*	2.1	1.44E-175	19384.62	40595.77
ENSG00000128872.10	*TMOD2*	2.1	1.02E-04	87.21	182.37
ENSG00000153071.15	*DAB2*	2.1	4.37E-14	497.60	1039.93
ENSG00000145817.17	*YIPF5*	2.1	3.61E-113	1063.59	2222.15
ENSG00000101230.6	*ISM1*	2.1	1.63E-21	142.45	297.25
ENSG00000168785.8	*TSPAN5*	2.1	1.67E-134	1328.06	2769.83
ENSG00000172465.14	*TCEAL1*	2.1	4.88E-47	284.12	591.98
ENSG00000125845.7	*BMP2*	2.1	1.03E-09	41.34	86.12
ENSG00000162772.17	*ATF3*	2.1	1.14E-07	81.53	169.70
ENSG00000185090.14	*MANEAL*	2.1	9.30E-04	86.62	180.03
ENSG00000134716.11	*CYP2J2*	2.1	6.44E-08	59.26	122.91
ENSG00000196975.16	*ANXA4*	2.1	1.14E-25	410.79	850.74
ENSG00000101882.10	*NKAP*	2.1	5.70E-26	276.17	571.92
ENSG00000164695.5	*CHMP4C*	2.1	2.03E-29	125.69	260.24
ENSG00000163754.17	*GYG1*	2.1	5.09E-87	552.47	1143.59
ENSG00000154529.14	*CNTNAP3B*	2.1	6.95E-03	25.59	52.95
ENSG00000205426.10	*KRT81*	2.1	4.02E-15	2520.77	5210.30
ENSG00000100478.15	*AP4S1*	2.1	2.64E-18	96.94	200.21
ENSG00000147394.18	*ZNF185*	2.1	2.05E-14	1398.86	2883.56
ENSG00000104356.11	*POP1*	2.1	9.44E-57	674.87	1390.37
ENSG00000110321.17	*EIF4G2*	2.1	1.21E-17	752.78	1550.73
ENSG00000164687.11	*FABP5*	2.1	1.58E-55	1287.48	2651.43
ENSG00000213523.10	*SRA1*	2.1	2.30E-17	225.96	465.22
ENSG00000226328.7	*NUP50-DT*	2.1	6.93E-21	83.50	171.89
ENSG00000102048.16	*ASB9*	2.1	4.54E-11	78.62	161.84
ENSG00000019549.13	*SNAI2*	2.1	2.88E-18	224.25	461.60
ENSG00000063978.16	*RNF4*	2.1	6.75E-87	2418.19	4966.31
ENSG00000166557.13	*TMED3*	2.1	6.12E-38	965.11	1978.60
ENSG00000263465.4	*SRSF8*	2.0	1.18E-11	57.53	117.83
ENSG00000124882.4	*EREG*	2.0	4.58E-08	315.91	646.88
ENSG00000135318.12	*NT5E*	2.0	7.51E-143	9389.45	19183.50
ENSG00000010810.17	*FYN*	2.0	8.00E-20	658.29	1344.94

ENSG00000156265.15	*MAP3K7CL*	2.0	3.48E-10	43.03	87.80
ENSG00000276116.2	*FUT8-AS1*	2.0	9.15E-04	24.20	49.37
ENSG00000138411.13	*HECW2*	2.0	1.81E-08	101.57	207.17
ENSG00000144959.10	*NCEH1*	2.0	7.66E-39	3686.05	7517.17
ENSG00000164743.5	*C8orf48*	2.0	1.26E-09	38.93	79.36
ENSG00000128610.12	*FEZF1*	2.0	3.62E-13	64.06	130.54
ENSG00000231991.4	*ANXA2P2*	2.0	8.27E-139	3010.25	6129.38
ENSG00000151414.15	*NEK7*	2.0	1.96E-106	2941.99	5976.50
ENSG00000261824.7	*LINC00662*	2.0	5.61E-12	157.80	320.47
ENSG00000074527.12	*NTN4*	2.0	9.53E-19	768.20	1560.15
ENSG00000165568.18	*AKR1E2*	2.0	1.28E-04	26.24	53.19
ENSG00000123575.9	*FAM199X*	2.0	4.45E-67	710.41	1439.85
ENSG00000155366.16	*RHOC*	2.0	1.03E-38	1278.47	2590.04
ENSG00000233461.6	*AL445524.1*	2.0	3.77E-07	91.51	185.25
ENSG00000110330.8	*BIRC2*	2.0	2.66E-07	189.73	384.01
ENSG00000104714.14	*ERICH1*	2.0	6.03E-33	212.53	429.95
ENSG00000230074.2	*AL162231.2*	2.0	3.35E-08	28.88	58.40
ENSG00000185761.11	*ADAMTSL5*	2.0	1.21E-22	171.67	347.06
ENSG00000102038.15	*SMARCA1*	2.0	4.75E-59	1211.95	2450.16
ENSG00000115310.18	*RTN4*	2.0	4.10E-135	8818.80	17807.40
ENSG00000204272.13	*NBDY*	2.0	6.18E-50	382.42	772.01
ENSG00000103942.13	*HOMER2*	2.0	1.06E-11	262.89	530.01
ENSG00000184860.10	*SDR42E1*	2.0	3.19E-29	271.03	545.71
ENSG00000140563.15	*MCTP2*	2.0	3.67E-03	32.58	65.60
ENSG00000253522.6	*MIR3142HG*	2.0	9.04E-06	23.89	47.98
ENSG00000171044.11	*XKR6*	2.0	5.54E-07	38.67	77.23
ENSG00000236349.1	*SUCLG2P2*	2.0	3.11E-11	64.75	129.27
ENSG00000124172.10	*ATP5F1E*	2.0	1.57E-117	2936.41	5859.01
ENSG00000175294.6	*CATSPER1*	2.0	9.72E-07	68.11	135.87
ENSG00000180530.11	*NRIP1*	2.0	6.76E-33	291.37	580.88
ENSG00000167315.18	*ACAA2*	-2.0	5.75E-37	854.49	428.55
ENSG00000115216.14	*NRBP1*	-2.0	5.53E-111	1747.73	875.63
ENSG00000139496.16	*NUP58*	-2.0	1.43E-85	1772.96	888.14
ENSG00000158201.10	*ABHD3*	-2.0	2.49E-32	360.38	180.49
ENSG00000176108.9	*CHMP6*	-2.0	1.58E-29	468.59	234.55
ENSG00000124787.14	*RPP40*	-2.0	5.80E-25	294.14	147.16
ENSG00000162545.6	*CAMK2N1*	-2.0	8.97E-32	571.12	285.74
ENSG00000153339.14	*TRAPPC8*	-2.0	2.54E-08	370.34	185.24
ENSG00000167291.16	*TBC1D16*	-2.0	6.59E-33	923.74	461.39
ENSG00000118705.17	*RPN2*	-2.0	4.49E-166	7334.62	3662.01
ENSG00000124615.20	*MOCS1*	-2.0	1.05E-31	667.35	333.05
ENSG00000077238.14	*IL4R*	-2.0	1.13E-28	1035.74	516.65
ENSG00000196367.13	*TRRAP*	-2.0	8.41E-10	1749.14	872.42
ENSG00000204435.13	*CSNK2B*	-2.0	4.30E-17	345.00	171.93

ENSG00000169100.14	*SLC25A6*	-2.0	3.17E-92	6648.11	3312.82
ENSG00000213626.13	*LBH*	-2.0	3.44E-09	142.82	71.11
ENSG00000175711.8	*B3GNTL1*	-2.0	3.76E-09	189.69	94.29
ENSG00000183828.15	*NUDT14*	-2.0	2.02E-21	226.72	112.69
ENSG00000176225.14	*RTTN*	-2.0	9.11E-26	368.15	182.94
ENSG00000198399.14	*ITSN2*	-2.0	1.13E-16	601.75	299.01
ENSG00000167113.11	*COQ4*	-2.0	1.51E-75	810.22	402.59
ENSG00000125257.16	*ABCC4*	-2.0	7.42E-35	892.53	443.46
ENSG00000139725.8	*RHOF*	-2.0	1.06E-36	998.57	496.11
ENSG00000163584.18	*RPL22L1*	-2.0	7.66E-67	1337.46	664.46
ENSG00000241404.7	*EGFL8*	-2.0	6.61E-06	103.31	51.32
ENSG00000173960.14	*UBXN2A*	-2.0	7.87E-55	544.07	269.91
ENSG00000055332.18	*EIF2AK2*	-2.0	1.32E-21	598.09	296.68
ENSG00000065328.16	*MCM10*	-2.0	9.50E-09	912.90	452.56
ENSG00000250565.7	*ATP6V1E2*	-2.0	4.13E-04	63.52	31.48
ENSG00000265688.2	*MAFG-DT*	-2.0	2.61E-07	93.31	46.25
ENSG00000146411.6	*SLC2A12*	-2.0	6.88E-08	62.97	31.20
ENSG00000204439.4	*C6orf47*	-2.0	1.96E-44	694.64	344.16
ENSG00000106610.15	*STAG3L4*	-2.0	5.63E-04	105.74	52.38
ENSG00000133026.12	*MYH10*	-2.0	8.02E-28	1890.85	936.63
ENSG00000081386.12	*ZNF510*	-2.0	3.00E-04	140.08	69.39
ENSG00000136100.14	*VPS36*	-2.0	2.94E-18	615.48	304.64
ENSG00000198938.2	*MT-CO3*	-2.0	9.26E-202	54972.32	27206.63
ENSG00000168758.11	*SEMA4C*	-2.0	1.35E-35	611.19	302.42
ENSG00000001617.12	*SEMA3F*	-2.0	1.10E-03	185.14	91.57
ENSG00000125971.16	*DYNLRB1*	-2.0	5.05E-10	115.35	57.02
ENSG00000104783.14	*KCNN4*	-2.0	1.00E-16	620.12	306.38
ENSG00000125375.15	*DMAC2L*	-2.0	3.44E-03	58.29	28.76
ENSG00000106683.15	*LIMK1*	-2.0	5.15E-112	1993.06	982.10
ENSG00000136153.20	*LMO7*	-2.0	2.46E-20	2619.58	1290.78
ENSG00000126858.18	*RHOT1*	-2.0	7.74E-09	310.09	152.76
ENSG00000075618.18	*FSCN1*	-2.0	2.20E-59	987.53	485.87
ENSG00000178498.16	*DTX3*	-2.0	3.14E-13	294.75	145.02
ENSG00000184226.15	*PCDH9*	-2.0	2.57E-06	83.57	41.07
ENSG00000064547.14	*LPAR2*	-2.0	1.20E-05	88.23	43.36
ENSG00000103196.12	*CRISPLD2*	-2.0	1.07E-08	93.28	45.84
ENSG00000035862.12	*TIMP2*	-2.0	1.47E-92	6760.84	3320.95
ENSG00000182389.19	*CACNB4*	-2.0	2.01E-03	103.29	50.73
ENSG00000163214.21	*DHX57*	-2.0	7.09E-16	391.40	192.22
ENSG00000100503.23	*NIN*	-2.0	4.15E-06	526.99	258.74
ENSG00000197020.11	*ZNF100*	-2.0	2.18E-07	222.04	109.01
ENSG00000198824.7	*CHAMP1*	-2.0	7.17E-22	683.98	335.82
ENSG00000136040.9	*PLXNC1*	-2.0	7.01E-05	51.77	25.41
ENSG00000115808.12	*STRN*	-2.0	6.59E-21	662.49	325.09

ENSG00000166228.9	*PCBD1*	-2.0	2.48E-60	901.18	442.08
ENSG00000115902.11	*SLC1A4*	-2.0	1.21E-16	180.18	88.35
ENSG00000114646.10	*CSPG5*	-2.0	9.10E-11	248.91	122.02
ENSG00000174721.10	*FGFBP3*	-2.0	2.50E-08	71.51	35.05
ENSG00000119787.14	*ATL2*	-2.0	3.38E-21	981.80	480.63
ENSG00000142657.21	*PGD*	-2.0	1.05E-125	3751.39	1835.14
ENSG00000185875.13	*THNSL1*	-2.0	1.41E-21	234.50	114.66
ENSG00000083642.19	*PDS5B*	-2.0	3.18E-44	800.09	391.12
ENSG00000079691.18	*CARMIL1*	-2.0	8.21E-08	241.43	117.91
ENSG00000151468.11	*CCDC3*	-2.0	1.51E-14	150.91	73.67
ENSG00000107821.14	*KAZALD1*	-2.0	1.75E-09	110.53	53.93
ENSG00000167106.12	*FAM102A*	-2.1	3.70E-06	837.94	408.54
ENSG00000082512.15	*TRAF5*	-2.1	1.14E-16	371.17	180.82
ENSG00000131480.9	*AOC2*	-2.1	4.11E-07	57.54	27.99
ENSG00000257594.4	*GALNT4*	-2.1	6.22E-21	314.41	152.78
ENSG00000101986.12	*ABCD1*	-2.1	8.44E-12	263.89	128.17
ENSG00000073350.13	*LLGL2*	-2.1	7.11E-03	76.05	36.93
ENSG00000124570.20	*SERPINB6*	-2.1	1.75E-24	958.90	465.13
ENSG00000064999.15	*ANKS1A*	-2.1	4.63E-80	770.36	373.44
ENSG00000165424.7	*ZCCHC24*	-2.1	1.22E-35	681.34	330.22
ENSG00000136144.12	*RCBTB1*	-2.1	3.90E-34	520.04	251.91
ENSG00000197008.9	*ZNF138*	-2.1	2.19E-05	85.16	41.25
ENSG00000163093.12	*BBS5*	-2.1	2.83E-10	129.11	62.53
ENSG00000198816.7	*ZNF358*	-2.1	9.59E-06	387.14	187.32
ENSG00000204371.11	*EHMT2*	-2.1	1.57E-05	584.43	282.59
ENSG00000136738.15	*STAM*	-2.1	3.05E-36	1055.99	509.71
ENSG00000174939.11	*ASPHD1*	-2.1	1.55E-12	223.87	108.05
ENSG00000166321.14	*NUDT13*	-2.1	8.73E-05	44.02	21.23
ENSG00000204438.11	*GPANK1*	-2.1	1.90E-39	458.99	221.26
ENSG00000214078.12	*CPNE1*	-2.1	5.20E-42	1497.89	721.53
ENSG00000141447.18	*OSBPL1A*	-2.1	2.46E-13	353.68	170.36
ENSG00000244045.13	*TMEM199*	-2.1	2.21E-18	496.59	239.08
ENSG00000123843.13	*C4BPB*	-2.1	3.48E-12	671.83	323.39
ENSG00000167703.14	*SLC43A2*	-2.1	5.41E-12	115.76	55.70
ENSG00000148484.18	*RSU1*	-2.1	1.61E-88	1645.87	791.94
ENSG00000080189.15	*SLC35C2*	-2.1	8.09E-50	875.20	420.63
ENSG00000089094.19	*KDM2B*	-2.1	2.57E-30	958.42	460.26
ENSG00000160094.15	*ZNF362*	-2.1	3.10E-15	219.52	105.37
ENSG00000257093.7	*DENND11*	-2.1	4.26E-77	753.68	361.75
ENSG00000165029.16	*ABCA1*	-2.1	3.33E-03	220.15	105.65
ENSG00000152457.18	*DCLRE1C*	-2.1	7.44E-12	367.74	176.47
ENSG00000196754.12	*S100A2*	-2.1	2.80E-17	574.91	275.87
ENSG00000148481.14	*MINDY3*	-2.1	8.00E-29	572.10	274.29
ENSG00000136754.17	*ABI1*	-2.1	1.45E-60	1196.06	573.28

ENSG00000169871.13	*TRIM56*	-2.1	2.90E-40	1583.31	758.77
ENSG00000221988.13	*PPT2*	-2.1	2.05E-51	708.31	339.31
ENSG00000143786.8	*CNIH3*	-2.1	2.22E-13	198.85	95.25
ENSG00000102710.20	*SUPT20H*	-2.1	6.38E-17	770.79	369.12
ENSG00000106066.15	*CPVL*	-2.1	1.53E-28	382.02	182.76
ENSG00000070961.15	*ATP2B1*	-2.1	7.10E-35	2071.96	991.02
ENSG00000143970.17	*ASXL2*	-2.1	2.63E-08	200.37	95.80
ENSG00000164855.16	*TMEM184A*	-2.1	4.47E-07	112.10	53.59
ENSG00000172466.16	*ZNF24*	-2.1	2.93E-79	1330.47	635.67
ENSG00000134463.15	*ECHDC3*	-2.1	7.21E-40	363.11	173.39
ENSG00000145029.13	*NICN1*	-2.1	4.39E-04	71.91	34.33
ENSG00000101439.9	*CST3*	-2.1	4.86E-77	4044.00	1930.64
ENSG00000198624.13	*CCDC69*	-2.1	3.82E-33	623.26	297.23
ENSG00000104964.14	*TLE5*	-2.1	2.73E-26	1348.99	643.25
ENSG00000169084.15	*DHRSX*	-2.1	2.81E-28	372.60	177.55
ENSG00000100034.14	*PPM1F*	-2.1	8.76E-44	2333.57	1109.28
ENSG00000150457.9	*LATS2*	-2.1	6.65E-65	812.93	386.33
ENSG00000196968.11	*FUT11*	-2.1	1.07E-29	349.26	165.90
ENSG00000015133.19	*CCDC88C*	-2.1	1.35E-24	687.45	326.38
ENSG00000070366.14	*SMG6*	-2.1	2.89E-33	1333.30	632.99
ENSG00000131187.9	*F12*	-2.1	2.22E-04	66.91	31.76
ENSG00000274070.2	*CASTOR2*	-2.1	1.26E-07	104.35	49.53
ENSG00000129103.18	*SUMF2*	-2.1	4.91E-20	998.63	473.71
ENSG00000203805.10	*PLPP4*	-2.1	4.43E-29	297.72	141.08
ENSG00000221926.12	*TRIM16*	-2.1	1.52E-42	732.96	347.26
ENSG00000137200.13	*CMTR1*	-2.1	7.68E-109	1876.28	888.70
ENSG00000139211.6	*AMIGO2*	-2.1	1.00E-04	502.22	237.77
ENSG00000151693.11	*ASAP2*	-2.1	1.00E-24	1258.43	595.31
ENSG00000124313.16	*IQSEC2*	-2.1	3.10E-08	393.35	185.99
ENSG00000154832.14	*CXXC1*	-2.1	2.10E-06	129.23	61.05
ENSG00000134330.19	*IAH1*	-2.1	5.95E-53	505.51	238.77
ENSG00000214530.9	*STARD10*	-2.1	7.79E-06	159.23	75.19
ENSG00000221821.4	*C6orf226*	-2.1	3.49E-10	102.93	48.60
ENSG00000164062.13	*APEH*	-2.1	3.96E-46	1648.21	778.18
ENSG00000120688.9	*WBP4*	-2.1	8.04E-47	431.29	203.59
ENSG00000100599.16	*RIN3*	-2.1	2.70E-40	480.35	226.60
ENSG00000152683.14	*SLC30A6*	-2.1	2.61E-56	698.67	329.44
ENSG00000143924.19	*EML4*	-2.1	2.87E-89	1431.12	674.29
ENSG00000160326.14	*SLC2A6*	-2.1	2.85E-20	290.65	136.84
ENSG00000169016.17	*E2F6*	-2.1	6.61E-24	386.56	181.99
ENSG00000170161.7	*AL512625.1*	-2.1	9.75E-06	81.15	38.20
ENSG00000171295.13	*ZNF440*	-2.1	8.17E-10	237.03	111.51
ENSG00000284024.2	*HSPA14*	-2.1	4.85E-13	168.61	79.32
ENSG00000196659.10	*TTC30B*	-2.1	3.19E-08	58.54	27.47

ENSG00000081059.20	*TCF7*	-2.1	2.08E-05	74.10	34.74
ENSG00000197467.14	*COL13A1*	-2.1	1.22E-14	972.40	455.78
ENSG00000148824.19	*MTG1*	-2.1	4.76E-45	480.16	225.02
ENSG00000171992.13	*SYNPO*	-2.1	9.75E-08	413.37	193.69
ENSG00000164307.13	*ERAP1*	-2.1	3.94E-40	1202.74	563.28
ENSG00000176438.12	*SYNE3*	-2.1	3.76E-12	248.95	116.36
ENSG00000124549.14	*BTN2A3P*	-2.1	3.65E-05	93.55	43.71
ENSG00000170954.11	*ZNF415*	-2.1	1.65E-11	137.31	64.15
ENSG00000143919.15	*CAMKMT*	-2.1	5.00E-06	70.93	33.10
ENSG00000115825.10	*PRKD3*	-2.1	2.93E-03	281.64	131.30
ENSG00000204564.12	*C6orf136*	-2.1	1.89E-28	446.02	207.92
ENSG00000253710.4	*ALG11*	-2.1	3.56E-08	431.17	200.87
ENSG00000121743.4	*GJA3*	-2.1	4.52E-15	192.24	89.55
ENSG00000135723.14	*FHOD1*	-2.1	1.59E-41	727.69	338.80
ENSG00000236104.3	*ZBTB22*	-2.1	1.11E-55	510.42	237.63
ENSG00000141627.13	*DYM*	-2.1	4.27E-10	329.60	153.45
ENSG00000167543.16	*TP53I13*	-2.1	1.24E-47	600.37	279.48
ENSG00000243406.6	*MRPS31P5*	-2.1	1.34E-04	70.12	32.64
ENSG00000143369.15	*ECM1*	-2.2	1.38E-55	728.94	338.42
ENSG00000081665.14	*ZNF506*	-2.2	3.59E-10	210.07	97.29
ENSG00000169372.13	*CRADD*	-2.2	3.77E-13	137.74	63.76
ENSG00000260917.1	*AL158212.3*	-2.2	6.33E-12	100.09	46.33
ENSG00000136044.12	*APPL2*	-2.2	5.22E-16	647.93	299.60
ENSG00000080839.12	*RBL1*	-2.2	2.18E-66	1230.94	569.04
ENSG00000151849.15	*CENPJ*	-2.2	1.06E-05	112.89	52.14
ENSG00000175906.5	*ARL4D*	-2.2	8.50E-16	247.02	114.08
ENSG00000091136.14	*LAMB1*	-2.2	1.94E-50	3509.32	1616.89
ENSG00000049323.16	*LTBP1*	-2.2	6.40E-23	684.10	314.77
ENSG00000100600.15	*LGMN*	-2.2	8.46E-72	730.41	335.82
ENSG00000213780.11	*GTF2H4*	-2.2	2.16E-23	692.81	318.13
ENSG00000183495.13	*EP400*	-2.2	1.69E-04	145.47	66.75
ENSG00000066084.13	*DIP2B*	-2.2	9.27E-29	1118.06	512.92
ENSG00000138073.14	*PREB*	-2.2	1.73E-80	1034.01	473.95
ENSG00000164068.16	*RNF123*	-2.2	3.89E-32	819.00	375.31
ENSG00000226137.5	*BAIAP2-DT*	-2.2	5.31E-11	128.47	58.85
ENSG00000136840.19	*ST6GALNAC4*	-2.2	2.44E-26	422.03	193.30
ENSG00000135452.10	*TSPAN31*	-2.2	2.46E-22	225.57	103.30
ENSG00000197457.10	*STMN3*	-2.2	2.91E-16	335.71	153.68
ENSG00000136715.18	*SAP130*	-2.2	2.16E-06	568.85	260.17
ENSG00000111145.8	*ELK3*	-2.2	9.30E-116	3566.41	1630.71
ENSG00000101452.15	*DHX35*	-2.2	1.26E-41	509.19	232.77
ENSG00000176170.13	*SPHK1*	-2.2	2.66E-10	236.12	107.87
ENSG00000228742.11	*LINC02577*	-2.2	5.70E-05	55.21	25.22
ENSG00000129219.14	*PLD2*	-2.2	5.20E-17	328.98	150.19

ENSG00000100982.12	*PCIF1*	-2.2	9.92E-77	855.79	390.66
ENSG00000120690.16	*ELF1*	-2.2	9.24E-56	623.78	284.66
ENSG00000115339.14	*GALNT3*	-2.2	4.98E-28	1281.66	584.20
ENSG00000151773.13	*CCDC122*	-2.2	1.36E-05	53.63	24.44
ENSG00000082212.13	*ME2*	-2.2	1.08E-115	1095.51	498.63
ENSG00000095397.14	*WHRN*	-2.2	2.19E-05	48.09	21.88
ENSG00000004975.12	*DVL2*	-2.2	1.82E-74	1159.00	527.32
ENSG00000135392.16	*DNAJC14*	-2.2	7.01E-03	88.24	40.13
ENSG00000108840.15	*HDAC5*	-2.2	9.60E-21	235.90	107.23
ENSG00000122033.14	*MTIF3*	-2.2	1.07E-07	128.18	58.24
ENSG00000101444.13	*AHCY*	-2.2	4.59E-206	4417.30	2005.91
ENSG00000108219.15	*TSPAN14*	-2.2	2.20E-35	588.70	267.07
ENSG00000151474.23	*FRMD4A*	-2.2	2.60E-13	767.04	347.83
ENSG00000162337.12	*LRP5*	-2.2	7.81E-53	2982.06	1351.71
ENSG00000197381.16	*ADARB1*	-2.2	2.77E-18	339.57	153.87
ENSG00000110888.17	*CAPRIN2*	-2.2	1.19E-13	739.79	335.09
ENSG00000141570.11	*CBX8*	-2.2	4.87E-41	346.78	157.07
ENSG00000121741.16	*ZMYM2*	-2.2	1.36E-07	402.82	182.39
ENSG00000198825.15	*INPP5F*	-2.2	7.64E-32	670.40	303.53
ENSG00000164898.13	*FMC1*	-2.2	3.29E-14	165.79	75.03
ENSG00000269416.6	*LINC01224*	-2.2	1.41E-05	122.95	55.58
ENSG00000213160.10	*KLHL23*	-2.2	8.50E-40	687.52	310.60
ENSG00000102595.20	*UGGT2*	-2.2	5.82E-20	296.15	133.68
ENSG00000159199.14	*ATP5MC1*	-2.2	6.07E-47	1669.44	753.28
ENSG00000010361.14	*FUZ*	-2.2	2.53E-05	42.35	19.11
ENSG00000149177.13	*PTPRJ*	-2.2	4.55E-35	1930.51	869.54
ENSG00000116525.14	*TRIM62*	-2.2	2.76E-10	141.31	63.60
ENSG00000269973.1	*AC010969.2*	-2.2	4.22E-13	95.67	43.03
ENSG00000269001.2	*AC092070.2*	-2.2	1.34E-03	82.31	37.01
ENSG00000152455.16	*SUV39H2*	-2.2	6.26E-56	745.25	334.99
ENSG00000115415.18	*STAT1*	-2.2	9.86E-11	234.53	105.42
ENSG00000124593.16	*AL365205.1*	-2.2	9.95E-25	249.50	112.13
ENSG00000167105.8	*TMEM92*	-2.2	4.17E-29	343.75	154.39
ENSG00000141458.13	*NPC1*	-2.2	7.53E-57	2069.58	929.17
ENSG00000141349.8	*G6PC3*	-2.2	3.06E-04	40.51	18.18
ENSG00000124177.15	*CHD6*	-2.2	7.80E-19	840.30	377.13
ENSG00000157240.4	*FZD1*	-2.2	1.79E-79	875.70	392.99
ENSG00000114315.4	*HES1*	-2.2	9.00E-20	241.31	108.13
ENSG00000008869.12	*HEATR5B*	-2.2	1.08E-36	462.89	207.41
ENSG00000237807.4	*AC022034.1*	-2.2	3.34E-20	482.67	216.20
ENSG00000108175.17	*ZMIZ1*	-2.2	5.23E-25	1993.61	892.66
ENSG00000156521.14	*TYSND1*	-2.2	1.01E-75	651.69	291.67
ENSG00000262664.3	*OVCA2*	-2.2	3.09E-75	589.83	263.53
ENSG00000166444.19	*ST5*	-2.2	1.70E-12	352.74	157.54

ENSG00000178467.18	*P4HTM*	-2.2	3.14E-31	426.98	190.68
ENSG00000144118.14	*RALB*	-2.2	5.14E-103	1111.92	495.95
ENSG00000112319.19	*EYA4*	-2.2	2.42E-05	108.64	48.45
ENSG00000124813.23	*RUNX2*	-2.2	6.87E-24	578.78	258.06
ENSG00000187607.16	*ZNF286A*	-2.2	1.63E-10	429.30	191.34
ENSG00000164045.12	*CDC25A*	-2.2	1.95E-51	1443.24	642.94
ENSG00000146776.14	*ATXN7L1*	-2.2	4.89E-04	41.34	18.41
ENSG00000173926.6	*RNF173*	-2.2	7.45E-20	302.70	134.60
ENSG00000102763.18	*VWA8*	-2.2	5.01E-14	335.57	149.18
ENSG00000164877.19	*MICALL2*	-2.3	2.43E-14	191.29	85.02
ENSG00000065413.20	*ANKRD44*	-2.3	1.01E-05	306.60	136.07
ENSG00000136720.7	*HS6ST1*	-2.3	1.72E-57	610.36	270.85
ENSG00000088179.9	*PTPN4*	-2.3	3.24E-04	231.08	102.52
ENSG00000196821.10	*ILRUN*	-2.3	1.66E-171	2657.01	1178.35
ENSG00000198888.2	*MT-ND1*	-2.3	9.62E-138	29684.46	13164.71
ENSG00000150477.15	*KIAA1328*	-2.3	3.35E-04	61.21	27.14
ENSG00000122861.16	*PLAU*	-2.3	1.23E-28	6238.88	2765.55
ENSG00000234072.1	*AC074117.1*	-2.3	1.19E-03	48.04	21.28
ENSG00000163517.15	*HDAC11*	-2.3	1.48E-07	139.27	61.70
ENSG00000130024.15	*PHF10*	-2.3	4.76E-51	1779.55	786.69
ENSG00000144026.12	*ZNF514*	-2.3	1.79E-18	366.66	162.03
ENSG00000104341.17	*LAPTM4B*	-2.3	3.88E-215	2832.22	1249.76
ENSG00000158715.6	*SLC45A3*	-2.3	2.08E-39	400.82	176.76
ENSG00000234608.8	*MAPKAPK5-AS1*	-2.3	2.65E-04	89.28	39.34
ENSG00000146856.14	*AGBL3*	-2.3	3.47E-05	55.11	24.28
ENSG00000112343.11	*TRIM38*	-2.3	1.32E-102	788.92	347.05
ENSG00000204348.10	*DXO*	-2.3	9.70E-34	276.03	121.34
ENSG00000107438.9	*PDLIM1*	-2.3	8.02E-60	1481.27	651.14
ENSG00000115042.10	*FAHD2A*	-2.3	3.31E-53	351.85	154.62
ENSG00000167721.10	*TSR1*	-2.3	2.53E-05	93.11	40.82
ENSG00000143375.15	*CGN*	-2.3	2.57E-03	65.48	28.71
ENSG00000171103.11	*TRMT61B*	-2.3	1.83E-20	152.24	66.73
ENSG00000181192.12	*DHTKD1*	-2.3	2.16E-65	505.56	221.53
ENSG00000153214.11	*TMEM87B*	-2.3	1.64E-22	1526.66	668.64
ENSG00000139722.7	*VPS37B*	-2.3	7.10E-199	1814.97	794.86
ENSG00000189050.16	*RNFT1*	-2.3	4.59E-14	146.96	64.34
ENSG00000108679.13	*LGALS3BP*	-2.3	1.81E-19	4894.51	2142.58
ENSG00000115525.18	*ST3GAL5*	-2.3	6.70E-10	96.53	42.25
ENSG00000196843.16	*ARID5A*	-2.3	1.32E-14	113.27	49.47
ENSG00000276045.3	*ORAI1*	-2.3	5.89E-39	795.65	347.39
ENSG00000123607.15	*TTC21B*	-2.3	1.86E-09	249.77	109.05
ENSG00000160447.7	*PKN3*	-2.3	1.26E-36	674.28	294.37
ENSG00000254635.6	*WAC-AS1*	-2.3	1.57E-37	352.89	153.99

ENSG00000134871.19	*COL4A2*	-2.3	1.58E-58	2908.88	1269.27
ENSG00000137434.11	*C6orf52*	-2.3	1.03E-06	71.77	31.25
ENSG00000204427.12	*ABHD16A*	-2.3	1.00E-72	788.44	343.25
ENSG00000159335.16	*PTMS*	-2.3	2.84E-07	417.28	181.56
ENSG00000267080.6	*ASB16-AS1*	-2.3	2.03E-12	111.53	48.52
ENSG00000184887.13	*BTBD6*	-2.3	2.86E-04	221.16	96.16
ENSG00000006025.12	*OSBPL7*	-2.3	2.17E-06	94.24	40.95
ENSG00000086730.17	*LAT2*	-2.3	1.20E-05	58.77	25.53
ENSG00000164056.11	*SPRY1*	-2.3	3.92E-59	675.66	293.55
ENSG00000175470.20	*PPP2R2D*	-2.3	8.11E-43	774.49	336.21
ENSG00000168283.14	*BMI1*	-2.3	2.54E-46	2155.64	930.77
ENSG00000137409.19	*MTCH1*	-2.3	8.94E-55	2569.41	1109.29
ENSG00000167889.12	*MGAT5B*	-2.3	9.41E-16	190.31	82.01
ENSG00000167118.10	*URM1*	-2.3	3.13E-25	629.89	271.09
ENSG00000187522.16	*HSPA14*	-2.3	4.55E-72	742.53	319.32
ENSG00000278619.5	*MRM1*	-2.3	9.12E-30	183.99	78.99
ENSG00000173581.7	*CCDC106*	-2.3	3.49E-15	215.93	92.55
ENSG00000106665.15	*CLIP2*	-2.3	2.22E-14	568.10	243.10
ENSG00000204386.11	*NEU1*	-2.3	5.99E-29	604.42	258.63
ENSG00000225697.13	*SLC26A6*	-2.3	6.69E-29	387.38	165.73
ENSG00000171132.14	*PRKCE*	-2.3	3.07E-08	500.19	213.82
ENSG00000099194.6	*SCD*	-2.3	1.78E-03	2494.32	1066.22
ENSG00000241058.4	*NSUN6*	-2.3	8.80E-42	280.85	119.84
ENSG00000154760.14	*SLFN13*	-2.3	2.25E-11	269.34	114.85
ENSG00000114541.15	*FRMD4B*	-2.3	2.20E-05	55.44	23.62
ENSG00000127863.15	*TNFRSF19*	-2.4	4.12E-25	161.42	68.68
ENSG00000152464.15	*RPP38*	-2.4	1.71E-48	350.37	149.06
ENSG00000136014.12	*USP44*	-2.4	1.98E-10	78.30	33.30
ENSG00000051825.15	*MPHOSPH9*	-2.4	5.76E-19	1068.05	453.94
ENSG00000139410.15	*SDSL*	-2.4	9.98E-28	311.53	132.30
ENSG00000269958.1	*AL049840.5*	-2.4	6.59E-15	115.63	49.05
ENSG00000107130.10	*NCS1*	-2.4	9.86E-43	1075.14	456.12
ENSG00000269190.6	*FBXO17*	-2.4	1.76E-29	436.11	184.85
ENSG00000181031.16	*RPH3AL*	-2.4	3.34E-35	1036.87	439.32
ENSG00000167363.14	*FN3K*	-2.4	4.91E-06	44.15	18.70
ENSG00000165572.8	*KBTBD6*	-2.4	3.90E-35	349.61	147.98
ENSG00000105963.15	*ADAP1*	-2.4	1.11E-08	68.60	29.01
ENSG00000133466.14	*C1QTNF6*	-2.4	8.75E-11	74.47	31.49
ENSG00000136867.11	*SLC31A2*	-2.4	2.19E-38	303.61	128.27
ENSG00000159314.11	*ARHGAP27*	-2.4	2.96E-19	426.99	180.28
ENSG00000124191.18	*TOX2*	-2.4	2.65E-41	367.61	155.01
ENSG00000139318.8	*DUSP6*	-2.4	2.74E-85	3209.68	1352.53
ENSG00000196700.9	*ZNF512B*	-2.4	6.38E-33	893.85	376.04
ENSG00000105520.10	*PLPPR2*	-2.4	1.08E-06	285.99	120.25

ENSG00000168237.18	*GLYCTK*	-2.4	6.01E-21	164.69	69.24
ENSG00000099998.17	*GGT5*	-2.4	4.91E-05	221.11	92.95
ENSG00000231528.3	*FAM225A*	-2.4	3.20E-10	610.73	256.55
ENSG00000213676.13	*ATF6B*	-2.4	4.49E-39	1687.26	708.68
ENSG00000248015.7	*AC005329.1*	-2.4	6.96E-07	51.95	21.81
ENSG00000186814.14	*ZSCAN30*	-2.4	1.42E-26	355.99	149.43
ENSG00000179403.12	*VWA1*	-2.4	1.84E-06	161.05	67.58
ENSG00000084444.14	*FAM234B*	-2.4	9.99E-28	201.77	84.65
ENSG00000169306.10	*IL1RAPL1*	-2.4	8.98E-13	130.28	54.66
ENSG00000125686.12	*MED1*	-2.4	1.12E-89	2523.74	1056.91
ENSG00000197808.12	*ZNF461*	-2.4	2.45E-04	76.14	31.89
ENSG00000138002.15	*IFT172*	-2.4	2.05E-03	108.76	45.54
ENSG00000075240.17	*GRAMD4*	-2.4	3.66E-12	269.26	112.57
ENSG00000173349.6	*SFT2D3*	-2.4	4.27E-18	108.43	45.33
ENSG00000062598.18	*ELMO2*	-2.4	1.73E-119	940.35	392.38
ENSG00000146678.10	*IGFBP1*	-2.4	2.34E-26	921.92	384.36
ENSG00000204304.12	*PBX2*	-2.4	1.81E-105	1366.00	568.78
ENSG00000220323.4	*HIST2H2BD*	-2.4	8.35E-03	63.65	26.44
ENSG00000160446.19	*ZDHHC12*	-2.4	3.19E-60	611.59	253.87
ENSG00000068724.17	*TTC7A*	-2.4	4.31E-19	401.38	166.56
ENSG00000100983.12	*GSS*	-2.4	1.08E-98	2711.28	1123.90
ENSG00000138030.13	*KHK*	-2.4	3.74E-16	166.90	69.17
ENSG00000143842.15	*SOX13*	-2.4	2.22E-66	737.62	305.68
ENSG00000204070.10	*SYS1*	-2.4	3.59E-26	859.61	356.09
ENSG00000003509.16	*NDUFAF7*	-2.4	4.78E-17	179.59	74.39
ENSG00000178531.6	*CTXN1*	-2.4	4.16E-14	324.29	134.31
ENSG00000103024.7	*NME3*	-2.4	5.06E-14	145.36	60.20
ENSG00000115760.14	*BIRC6*	-2.4	7.02E-08	452.24	187.05
ENSG00000164088.18	*PPM1M*	-2.4	4.43E-37	267.22	110.45
ENSG00000008277.14	*ADAM22*	-2.4	7.43E-05	105.85	43.75
ENSG00000100065.15	*CARD10*	-2.4	6.03E-52	672.18	277.73
ENSG00000152518.8	*ZFP36L2*	-2.4	4.49E-100	828.29	342.17
ENSG00000115109.14	*EPB41L5*	-2.4	2.93E-17	267.16	110.32
ENSG00000133104.14	*SPART*	-2.4	9.42E-73	1094.01	451.67
ENSG00000183018.9	*SPNS2*	-2.4	6.33E-04	122.08	50.37
ENSG00000101040.19	*ZMYND8*	-2.4	2.43E-29	1414.90	583.76
ENSG00000145246.14	*ATP10D*	-2.4	1.49E-16	921.65	379.86
ENSG00000181513.14	*ACBD4*	-2.4	1.36E-06	61.87	25.49
ENSG00000234444.10	*ZNF736*	-2.4	7.56E-11	249.15	102.59
ENSG00000092010.15	*PSME1*	-2.4	4.07E-121	1907.26	785.08
ENSG00000142920.17	*AZIN2*	-2.4	1.70E-10	170.80	70.30
ENSG00000124496.12	*TRERF1*	-2.4	1.16E-53	1227.78	505.29
ENSG00000119686.10	*FLVCR2*	-2.4	1.37E-05	63.32	26.05
ENSG00000230795.3	*HLA-K*	-2.4	1.01E-12	80.70	33.19

ENSG00000173894.11	*CBX2*	-2.4	3.29E-26	503.09	206.87
ENSG00000270504.1	*AL391422.4*	-2.4	4.16E-11	91.01	37.42
ENSG00000269940.1	*AL049840.4*	-2.4	5.78E-14	78.32	32.18
ENSG00000286177.1	*AC011462.5*	-2.4	1.80E-09	54.49	22.38
ENSG00000105518.14	*TMEM205*	-2.4	1.62E-76	568.25	233.37
ENSG00000078403.17	*MLLT10*	-2.4	4.73E-15	571.98	234.57
ENSG00000068001.14	*HYAL2*	-2.4	1.08E-73	809.46	331.31
ENSG00000106789.13	*CORO2A*	-2.4	1.51E-30	499.48	204.40
ENSG00000120832.10	*MTERF2*	-2.4	2.86E-17	129.84	53.09
ENSG00000153933.10	*DGKE*	-2.4	2.22E-06	47.76	19.53
ENSG00000167272.11	*POP5*	-2.4	1.77E-43	548.62	224.24
ENSG00000112139.16	*MDGA1*	-2.4	1.80E-04	60.11	24.55
ENSG00000010256.11	*UQCRC1*	-2.5	1.29E-178	3620.24	1476.34
ENSG00000127720.8	*METTL25*	-2.5	1.66E-06	64.67	26.32
ENSG00000167393.18	*PPP2R3B*	-2.5	3.67E-42	341.41	138.61
ENSG00000256028.2	*AC026362.1*	-2.5	6.25E-05	90.08	36.56
ENSG00000197892.13	*KIF13B*	-2.5	3.18E-69	701.46	284.59
ENSG00000121653.11	*MAPK8IP1*	-2.5	2.21E-34	286.48	116.22
ENSG00000204590.12	*GNL1*	-2.5	1.49E-22	321.87	130.50
ENSG00000240489.1	*SETP14*	-2.5	4.92E-11	132.94	53.89
ENSG00000272462.3	*U91328.1*	-2.5	5.34E-07	51.55	20.89
ENSG00000169126.16	*ARMC4*	-2.5	1.45E-08	135.32	54.81
ENSG00000090975.12	*PITPNM2*	-2.5	3.47E-05	149.03	60.28
ENSG00000102531.16	*FNDC3A*	-2.5	9.74E-88	1164.04	470.74
ENSG00000155254.13	*MARVELD1*	-2.5	9.16E-49	690.55	279.21
ENSG00000146950.13	*SHROOM2*	-2.5	4.74E-08	326.22	131.88
ENSG00000277534.1	*AC007996.1*	-2.5	1.86E-07	53.37	21.56
ENSG00000101363.12	*MANBAL*	-2.5	5.62E-40	477.38	192.75
ENSG00000115756.13	*HPCAL1*	-2.5	1.06E-145	3279.53	1321.51
ENSG00000078967.13	*UBE2D4*	-2.5	6.54E-40	370.99	149.37
ENSG00000149639.15	*SOGA1*	-2.5	2.42E-30	1866.31	749.78
ENSG00000154914.17	*USP43*	-2.5	1.00E-13	103.35	41.51
ENSG00000169174.10	*PCSK9*	-2.5	1.27E-03	203.45	81.67
ENSG00000176155.19	*CCDC57*	-2.5	4.34E-15	346.96	139.14
ENSG00000115207.13	*GTF3C2*	-2.5	2.48E-10	85.03	34.05
ENSG00000081803.16	*CADPS2*	-2.5	1.13E-36	272.76	109.20
ENSG00000152242.11	*C18orf25*	-2.5	3.08E-21	677.18	270.72
ENSG00000213689.14	*TREX1*	-2.5	6.61E-13	119.77	47.88
ENSG00000183891.6	*TTC32*	-2.5	2.51E-09	64.82	25.84
ENSG00000184922.14	*FMNL1*	-2.5	2.86E-42	2107.84	840.19
ENSG00000181284.3	*TMEM102*	-2.5	5.88E-10	51.98	20.72
ENSG00000136758.18	*YME1L1*	-2.5	3.72E-25	1374.65	547.73
ENSG00000166974.13	*MAPRE2*	-2.5	7.97E-27	646.67	257.55
ENSG00000169239.13	*CA5B*	-2.5	2.76E-09	170.13	67.75

ENSG00000205336.12	*ADGRG1*	-2.5	1.48E-30	485.24	193.19
ENSG00000127920.6	*GNG11*	-2.5	2.70E-39	443.31	176.46
ENSG00000054690.14	*PLEKHH1*	-2.5	9.57E-06	94.39	37.56
ENSG00000106211.9	*HSPB1*	-2.5	4.76E-23	2590.81	1030.62
ENSG00000137413.16	*TAF8*	-2.5	1.49E-16	449.76	178.84
ENSG00000008311.15	*AASS*	-2.5	2.23E-46	756.33	300.58
ENSG00000173041.12	*ZNF680*	-2.5	6.40E-23	201.63	80.12
ENSG00000126001.16	*CEP250*	-2.5	9.88E-64	2654.79	1054.30
ENSG00000105376.5	*ICAM5*	-2.5	3.22E-06	51.05	20.26
ENSG00000218739.10	*CEBPZOS*	-2.5	4.28E-51	506.95	201.05
ENSG00000124181.14	*PLCG1*	-2.5	7.39E-53	2036.32	807.34
ENSG00000169247.12	*SH3TC2*	-2.5	1.77E-09	403.16	159.51
ENSG00000175866.15	*BAIAP2*	-2.5	3.27E-36	986.32	390.18
ENSG00000250264.1	*AL669918.1*	-2.5	1.92E-04	107.84	42.61
ENSG00000151694.14	*ADAM17*	-2.5	2.99E-47	645.37	255.02
ENSG00000161513.12	*FDXR*	-2.5	2.93E-20	128.79	50.89
ENSG00000185989.11	*RASA3*	-2.5	7.96E-159	1304.82	515.16
ENSG00000187244.11	*BCAM*	-2.5	3.94E-08	172.39	68.04
ENSG00000065060.17	*UHRF1BP1*	-2.5	2.95E-64	1052.38	415.27
ENSG00000168394.11	*TAP1*	-2.5	3.53E-03	155.33	61.24
ENSG00000234745.11	*HLA-B*	-2.5	2.49E-22	2794.78	1099.78
ENSG00000172819.17	*RARG*	-2.5	4.58E-23	739.39	290.36
ENSG00000152527.14	*PLEKHH2*	-2.5	1.65E-03	60.25	23.65
ENSG00000101104.12	*PABPC1L*	-2.6	9.50E-16	241.25	94.36
ENSG00000198521.11	*ZNF43*	-2.6	8.87E-09	164.86	64.48
ENSG00000136152.15	*COG3*	-2.6	2.63E-16	459.33	179.64
ENSG00000160404.18	*TOR2A*	-2.6	4.06E-29	271.04	105.97
ENSG00000120662.16	*MTRF1*	-2.6	4.34E-15	95.05	37.14
ENSG00000130775.16	*THEMIS2*	-2.6	3.26E-07	88.31	34.51
ENSG00000189319.14	*FAM53B*	-2.6	8.58E-59	453.80	177.24
ENSG00000152240.13	*HAUS1*	-2.6	8.69E-61	801.11	312.86
ENSG00000127946.17	*HIP1*	-2.6	7.83E-13	315.03	123.03
ENSG00000089159.16	*PXN*	-2.6	7.74E-127	8948.98	3494.63
ENSG00000130158.14	*DOCK6*	-2.6	4.66E-08	125.18	48.75
ENSG00000096070.19	*BRPF3*	-2.6	2.18E-83	2890.22	1124.40
ENSG00000184988.8	*TMEM106A*	-2.6	7.91E-08	52.88	20.54
ENSG00000130005.13	*GAMT*	-2.6	2.07E-126	1109.19	430.71
ENSG00000099256.19	*PRTFDC1*	-2.6	2.72E-47	367.65	142.73
ENSG00000167705.12	*RILP*	-2.6	1.25E-24	207.53	80.57
ENSG00000112541.15	*PDE10A*	-2.6	8.69E-06	273.40	106.09
ENSG00000171159.5	*C9orf16*	-2.6	2.15E-46	271.50	105.13
ENSG00000084112.15	*SSH1*	-2.6	3.87E-43	2449.75	948.40
ENSG00000163513.18	*TGFBR2*	-2.6	2.20E-22	1179.08	455.67
ENSG00000138018.18	*SELENOI*	-2.6	9.28E-67	1477.94	571.08

ENSG00000171448.9	*ZBTB26*	-2.6	5.01E-17	149.35	57.70
ENSG00000126005.17	*MMP24OS*	-2.6	4.78E-95	1547.67	597.50
ENSG00000226479.4	*TMEM185B*	-2.6	2.80E-110	1026.89	396.40
ENSG00000149260.18	*CAPN5*	-2.6	3.20E-22	296.58	114.40
ENSG00000248323.7	*LUCAT1*	-2.6	1.51E-09	114.85	44.28
ENSG00000124151.19	*NCOA3*	-2.6	3.07E-43	786.09	302.99
ENSG00000167716.18	*WDR81*	-2.6	3.06E-04	97.68	37.61
ENSG00000165886.5	*UBTD1*	-2.6	1.88E-94	604.92	232.85
ENSG00000163798.13	*SLC4A1AP*	-2.6	9.72E-10	283.09	108.92
ENSG00000171017.11	*LRRC8E*	-2.6	2.89E-24	278.78	107.17
ENSG00000225684.4	*FAM225B*	-2.6	7.16E-23	457.60	175.88
ENSG00000135469.13	*COQ10A*	-2.6	5.41E-12	147.45	56.67
ENSG00000272121.1	*AC006058.3*	-2.6	2.06E-11	67.50	25.94
ENSG00000157693.15	*TMEM268*	-2.6	2.18E-14	157.47	60.40
ENSG00000164078.13	*MST1R*	-2.6	2.47E-13	256.69	98.45
ENSG00000095787.23	*WAC*	-2.6	2.37E-76	1769.22	678.25
ENSG00000143740.14	*SNAP47*	-2.6	1.47E-05	45.77	17.53
ENSG00000125347.14	*IRF1*	-2.6	3.32E-25	475.07	181.89
ENSG00000141753.7	*IGFBP4*	-2.6	3.78E-114	29348.26	11202.02
ENSG00000175087.10	*PDIK1L*	-2.6	1.50E-17	248.23	94.66
ENSG00000150867.14	*PIP4K2A*	-2.6	1.94E-51	1607.20	612.40
ENSG00000187498.16	*COL4A1*	-2.6	7.74E-36	915.78	348.87
ENSG00000106125.14	*MINDY4*	-2.6	4.27E-11	79.16	30.14
ENSG00000164048.14	*ZNF589*	-2.6	1.45E-18	164.66	62.68
ENSG00000078142.13	*PIK3C3*	-2.6	6.75E-43	840.93	320.06
ENSG00000257524.6	*AL157935.2*	-2.6	7.33E-05	65.72	25.00
ENSG00000005810.18	*MYCBP2*	-2.6	7.00E-13	468.60	178.06
ENSG00000106049.9	*HIBADH*	-2.6	6.99E-83	910.12	345.43
ENSG00000204525.16	*HLA-C*	-2.6	7.27E-36	669.59	253.81
ENSG00000259953.2	*AL138756.1*	-2.6	1.00E-14	77.05	29.19
ENSG00000139178.11	*C1RL*	-2.6	5.32E-05	44.20	16.75
ENSG00000068650.18	*ATP11A*	-2.6	5.86E-16	577.13	218.46
ENSG00000120539.14	*MASTL*	-2.6	9.62E-52	1127.69	426.11
ENSG00000182054.9	*IDH2*	-2.6	8.69E-10	137.55	51.96
ENSG00000186787.8	*SPIN2B*	-2.6	8.91E-03	127.91	48.28
ENSG00000196187.12	*TMEM63A*	-2.7	6.07E-73	654.36	246.92
ENSG00000164171.11	*ITGA2*	-2.7	8.06E-78	6010.36	2266.67
ENSG00000172059.11	*KLF11*	-2.7	1.60E-23	229.50	86.55
ENSG00000172766.19	*NAA16*	-2.7	6.35E-22	302.79	114.18
ENSG00000088387.19	*DOCK9*	-2.7	3.43E-16	381.99	143.80
ENSG00000134987.11	*WDR36*	-2.7	3.74E-07	59.05	22.23
ENSG00000169598.15	*DFFB*	-2.7	6.41E-04	67.63	25.41
ENSG00000153208.17	*MERTK*	-2.7	8.57E-69	728.99	273.66
ENSG00000155363.18	*MOV10*	-2.7	1.04E-08	182.89	68.59

ENSG00000107816.17	*LZTS2*	-2.7	8.62E-08	313.18	117.22
ENSG00000215039.7	*CD27-AS1*	-2.7	5.55E-15	109.18	40.82
ENSG00000169026.12	*SLC49A3*	-2.7	4.84E-05	48.03	17.94
ENSG00000276672.1	*AL161891.1*	-2.7	8.47E-32	205.43	76.62
ENSG00000134574.11	*DDB2*	-2.7	1.07E-05	139.36	51.92
ENSG00000141452.10	*RMC1*	-2.7	4.72E-52	530.97	197.58
ENSG00000178922.16	*HYI*	-2.7	9.62E-34	1113.48	413.44
ENSG00000130304.17	*SLC27A1*	-2.7	2.79E-27	168.33	62.32
ENSG00000213096.10	*ZNF254*	-2.7	7.01E-04	46.57	17.24
ENSG00000027001.10	*MIPEP*	-2.7	2.64E-41	255.79	94.49
ENSG00000166166.13	*TRMT61A*	-2.7	1.65E-128	789.75	291.66
ENSG00000170689.10	*HOXB9*	-2.7	5.69E-43	211.77	77.91
ENSG00000006062.17	*MAP3K14*	-2.7	8.75E-88	825.21	303.22
ENSG00000092929.11	*UNC13D*	-2.7	5.76E-18	283.73	104.25
ENSG00000178695.6	*KCTD12*	-2.7	1.41E-152	1171.01	430.18
ENSG00000107551.21	*RASSF4*	-2.7	9.14E-15	193.55	70.88
ENSG00000129422.14	*MTUS1*	-2.7	1.93E-05	114.79	41.96
ENSG00000136717.15	*BIN1*	-2.7	1.40E-29	765.99	279.79
ENSG00000156411.9	*ATP5MPL*	-2.7	7.98E-08	83.69	30.48
ENSG00000137166.15	*FOXP4*	-2.7	2.11E-101	1597.37	581.27
ENSG00000147676.14	*MAL2*	-2.8	4.95E-45	587.44	213.38
ENSG00000219626.9	*FAM228B*	-2.8	4.31E-05	40.99	14.88
ENSG00000140931.20	*CMTM3*	-2.8	1.20E-33	283.81	102.97
ENSG00000168765.17	*GSTM4*	-2.8	1.12E-35	249.72	90.38
ENSG00000157657.14	*ZNF618*	-2.8	6.49E-07	257.12	93.01
ENSG00000100711.13	*ZFYVE21*	-2.8	8.69E-13	146.30	52.92
ENSG00000150051.14	*MKX*	-2.8	2.00E-25	122.44	44.18
ENSG00000107331.17	*ABCA2*	-2.8	1.10E-38	544.12	196.24
ENSG00000184602.6	*SNN*	-2.8	6.92E-18	146.49	52.82
ENSG00000213722.9	*DDAH2*	-2.8	1.82E-51	1031.70	371.76
ENSG00000175782.10	*SLC35E3*	-2.8	7.90E-06	79.16	28.35
ENSG00000134324.11	*LPIN1*	-2.8	3.37E-11	582.08	208.38
ENSG00000074219.13	*TEAD2*	-2.8	8.69E-18	343.31	122.90
ENSG00000165752.17	*STK32C*	-2.8	2.52E-96	646.02	231.07
ENSG00000079263.19	*SP140*	-2.8	3.30E-20	127.98	45.75
ENSG00000133101.10	*CCNA1*	-2.8	8.54E-22	253.44	90.58
ENSG00000075413.18	*MARK3*	-2.8	1.83E-31	752.94	268.95
ENSG00000126759.13	*CFP*	-2.8	1.02E-06	217.45	77.47
ENSG00000153246.13	*PLA2R1*	-2.8	1.65E-05	65.92	23.47
ENSG00000163710.9	*PCOLCE2*	-2.8	1.36E-09	73.90	26.22
ENSG00000164841.5	*TMEM74*	-2.8	2.56E-12	65.76	23.28
ENSG00000106003.13	*LFNG*	-2.8	2.41E-21	330.53	116.80
ENSG00000123689.6	*G0S2*	-2.8	2.15E-57	2348.27	828.52
ENSG00000228223.3	*HCG11*	-2.8	4.03E-05	58.08	20.47

ENSG00000233493.4	*TMEM238*	-2.8	2.77E-15	72.64	25.57
ENSG00000124225.16	*PMEPA1*	-2.8	1.34E-10	982.10	345.10
ENSG00000169629.12	*RGPD8*	-2.8	1.31E-08	253.29	88.96
ENSG00000179630.11	*LACC1*	-2.8	1.50E-21	149.24	52.39
ENSG00000124104.19	*SNX21*	-2.9	6.54E-26	368.53	129.28
ENSG00000163701.19	*IL17RE*	-2.9	3.36E-11	103.12	36.15
ENSG00000138101.18	*DTNB*	-2.9	1.43E-06	65.60	22.99
ENSG00000112769.20	*LAMA4*	-2.9	1.02E-03	58.80	20.56
ENSG00000137203.12	*TFAP2A*	-2.9	1.22E-04	59.02	20.61
ENSG00000107614.22	*TRDMT1*	-2.9	2.43E-18	269.47	94.00
ENSG00000138074.15	*SLC5A6*	-2.9	9.32E-56	736.35	256.41
ENSG00000112576.12	*CCND3*	-2.9	4.13E-33	446.22	155.36
ENSG00000121797.10	*CCRL2*	-2.9	2.13E-56	447.17	155.66
ENSG00000084676.15	*NCOA1*	-2.9	3.69E-12	250.49	86.89
ENSG00000152234.16	*ATP5F1A*	-2.9	0.00E+00	9565.03	3314.74
ENSG00000160991.16	*ORAI2*	-2.9	1.53E-03	436.56	151.11
ENSG00000083544.14	*TDRD3*	-2.9	4.01E-07	157.74	54.57
ENSG00000267368.1	*UPK3BL1*	-2.9	3.19E-04	96.11	33.21
ENSG00000198223.16	*CSF2RA*	-2.9	1.06E-03	154.63	53.43
ENSG00000178150.10	*ZNF114*	-2.9	2.92E-20	822.74	284.20
ENSG00000164136.17	*IL15*	-2.9	2.33E-11	56.08	19.32
ENSG00000057704.13	*TMCC3*	-2.9	1.57E-38	351.62	120.90
ENSG00000171222.10	*SCAND1*	-2.9	5.16E-19	141.20	48.50
ENSG00000168016.14	*TRANK1*	-2.9	2.11E-04	236.99	81.40
ENSG00000167123.19	*CERCAM*	-2.9	7.34E-61	698.54	238.52
ENSG00000206341.7	*HLA-H*	-2.9	1.32E-44	408.59	139.37
ENSG00000156966.7	*B3GNT7*	-2.9	2.09E-05	120.21	40.85
ENSG00000119514.7	*GALNT12*	-2.9	4.77E-07	50.60	17.19
ENSG00000118777.12	*ABCG2*	-2.9	1.07E-35	321.12	109.07
ENSG00000107897.19	*ACBD5*	-2.9	4.23E-153	1001.13	339.90
ENSG00000152223.15	*EPG5*	-3.0	4.11E-03	678.51	229.67
ENSG00000178773.15	*CPNE7*	-3.0	9.15E-06	70.88	23.98
ENSG00000105514.8	*RAB3D*	-3.0	1.36E-15	73.68	24.90
ENSG00000179583.19	*CIITA*	-3.0	7.10E-03	40.16	13.56
ENSG00000106948.16	*AKNA*	-3.0	1.36E-61	380.44	128.28
ENSG00000105767.3	*CADM4*	-3.0	3.38E-10	43.78	14.71
ENSG00000107968.10	*MAP3K8*	-3.0	3.90E-11	67.26	22.56
ENSG00000204366.4	*ZBTB12*	-3.0	6.88E-38	257.18	86.18
ENSG00000164850.15	*GPER1*	-3.0	1.39E-13	71.48	23.94
ENSG00000154930.15	*ACSS1*	-3.0	1.07E-19	161.03	53.88
ENSG00000137834.15	*SMAD6*	-3.0	5.90E-06	65.78	21.94
ENSG00000126214.21	*KLC1*	-3.0	5.26E-72	2024.40	675.23
ENSG00000137198.10	*GMPR*	-3.0	3.90E-66	401.55	133.76
ENSG00000188483.8	*IER5L*	-3.0	5.63E-24	332.27	110.36

ENSG00000154065.17	*ANKRD29*	-3.0	2.04E-25	184.12	61.13
ENSG00000148468.17	*FAM171A1*	-3.0	4.40E-102	1041.37	345.75
ENSG00000125968.9	*ID1*	-3.0	1.58E-43	2085.23	691.80
ENSG00000180573.9	*HIST1H2AC*	-3.0	3.96E-08	148.91	49.40
ENSG00000198804.2	*MT-CO1*	-3.0	0.00E+00	131545.70	43623.99
ENSG00000123342.16	*MMP19*	-3.0	2.26E-11	56.60	18.72
ENSG00000142910.16	*TINAGL1*	-3.0	9.78E-78	1490.87	492.87
ENSG00000163596.16	*ICA1L*	-3.0	5.61E-04	44.47	14.69
ENSG00000139793.18	*MBNL2*	-3.0	3.77E-15	210.93	69.58
ENSG00000090376.11	*IRAK3*	-3.0	1.03E-22	115.28	38.01
ENSG00000147650.11	*LRP12*	-3.0	4.67E-18	913.49	300.42
ENSG00000165997.5	*ARL5B*	-3.0	7.86E-91	868.86	285.19
ENSG00000115295.20	*CLIP4*	-3.0	3.00E-08	281.47	92.36
ENSG00000126215.14	*XRCC3*	-3.0	8.40E-72	961.07	315.15
ENSG00000163026.12	*WDCP*	-3.1	1.19E-32	339.58	111.28
ENSG00000165474.8	*GJB2*	-3.1	2.43E-55	324.05	106.13
ENSG00000287431.1	*AC027601.6*	-3.1	1.32E-07	43.51	14.23
ENSG00000179085.7	*DPM3*	-3.1	1.67E-13	72.15	23.55
ENSG00000076351.13	*SLC46A1*	-3.1	1.89E-17	424.87	138.56
ENSG00000286132.1	*AC022415.2*	-3.1	3.27E-28	156.27	50.96
ENSG00000111275.13	*ALDH2*	-3.1	6.48E-109	1346.89	437.88
ENSG00000144485.11	*HES6*	-3.1	1.08E-19	123.86	40.24
ENSG00000171813.14	*PWWP2B*	-3.1	5.92E-58	341.87	111.05
ENSG00000012171.20	*SEMA3B*	-3.1	2.95E-10	365.24	118.64
ENSG00000280120.1	*AC073857.1*	-3.1	3.72E-14	59.16	19.17
ENSG00000176244.7	*ACBD7*	-3.1	4.16E-21	160.66	52.05
ENSG00000156026.14	*MCU*	-3.1	1.97E-03	55.69	17.97
ENSG00000165996.14	*HACD1*	-3.1	2.59E-111	567.89	183.11
ENSG00000196814.15	*MVB12B*	-3.1	1.33E-17	117.36	37.68
ENSG00000130940.15	*CASZ1*	-3.1	1.24E-07	130.51	41.90
ENSG00000136244.12	*IL6*	-3.1	1.31E-05	40.53	13.00
ENSG00000173193.15	*PARP14*	-3.1	6.59E-16	460.09	147.14
ENSG00000105497.8	*ZNF175*	-3.1	5.56E-16	466.32	149.13
ENSG00000197283.17	*SYNGAP1*	-3.1	7.81E-03	69.30	22.14
ENSG00000124664.11	*SPDEF*	-3.1	7.73E-23	623.37	198.33
ENSG00000177706.9	*FAM20C*	-3.1	7.29E-95	695.13	220.92
ENSG00000121454.6	*LHX4*	-3.1	5.43E-29	128.39	40.78
ENSG00000237973.1	*MTCO1P12*	-3.1	2.97E-182	3241.10	1028.96
ENSG00000176595.4	*KBTBD11*	-3.2	1.86E-19	138.65	43.73
ENSG00000171462.15	*DLK2*	-3.2	6.16E-15	92.14	29.01
ENSG00000233237.8	*LINC00472*	-3.2	1.43E-73	801.03	251.71
ENSG00000133135.14	*RNF128*	-3.2	5.83E-13	54.66	17.17
ENSG00000059378.12	*PARP12*	-3.2	2.59E-05	72.04	22.58
ENSG00000147408.14	*CSGALNACT1*	-3.2	8.75E-03	53.18	16.66

ENSG00000148344.11	*PTGES*	-3.2	2.97E-16	424.42	132.28
ENSG00000175832.13	*ETV4*	-3.2	2.80E-231	1168.35	362.26
ENSG00000267121.6	*AC008105.3*	-3.2	4.08E-11	68.60	21.22
ENSG00000169129.15	*AFAP1L2*	-3.3	1.09E-26	785.13	241.53
ENSG00000164920.9	*OSR2*	-3.3	8.41E-03	60.90	18.66
ENSG00000158373.8	*HIST1H2BD*	-3.3	5.94E-10	74.76	22.86
ENSG00000111684.11	*LPCAT3*	-3.3	1.55E-32	404.08	123.45
ENSG00000175591.11	*P2RY2*	-3.3	9.55E-27	300.28	91.51
ENSG00000102760.13	*RGCC*	-3.3	5.14E-09	53.13	16.19
ENSG00000196535.16	*MYO18A*	-3.3	1.53E-41	1022.76	310.88
ENSG00000117676.14	*RPS6KA1*	-3.3	2.67E-84	874.48	265.62
ENSG00000126351.12	*THRA*	-3.3	2.48E-04	44.67	13.55
ENSG00000217801.10	*AL390719.1*	-3.3	2.09E-07	59.88	18.13
ENSG00000158106.14	*RHPN1*	-3.3	1.07E-12	123.49	37.19
ENSG00000082014.16	*SMARCD3*	-3.3	2.44E-03	41.85	12.58
ENSG00000078699.21	*CBFA2T2*	-3.3	8.35E-05	84.30	25.31
ENSG00000182704.8	*TSKU*	-3.3	6.44E-60	522.37	156.24
ENSG00000278535.5	*DHRS11*	-3.3	7.18E-44	303.65	90.82
ENSG00000163694.15	*RBM47*	-3.3	2.03E-15	144.18	43.12
ENSG00000215146.5	*BX322639.1*	-3.3	2.77E-17	100.50	30.03
ENSG00000167880.7	*EVPL*	-3.4	4.81E-05	191.35	57.03
ENSG00000285796.1	*AL162458.1*	-3.4	1.16E-07	44.20	13.16
ENSG00000099204.20	*ABLIM1*	-3.4	6.42E-154	2384.98	708.87
ENSG00000138095.19	*LRPPRC*	-3.4	2.18E-37	431.50	127.66
ENSG00000185187.13	*SIGIRR*	-3.4	9.02E-08	102.90	30.43
ENSG00000266208.1	*AC080112.1*	-3.4	1.70E-09	65.38	19.29
ENSG00000029534.20	*ANK1*	-3.4	9.64E-11	192.40	56.47
ENSG00000026950.17	*BTN3A1*	-3.4	2.37E-15	204.88	60.02
ENSG00000124006.15	*OBSL1*	-3.4	5.26E-12	558.99	163.47
ENSG00000174307.7	*PHLDA3*	-3.4	2.30E-14	235.55	68.57
ENSG00000174705.13	*SH3PXD2B*	-3.4	2.31E-24	173.43	50.48
ENSG00000177409.12	*SAMD9L*	-3.5	4.03E-14	366.76	105.89
ENSG00000163898.10	*LIPH*	-3.5	3.14E-14	108.79	31.40
ENSG00000214189.9	*ZNF788P*	-3.5	2.86E-42	196.02	56.50
ENSG00000109103.11	*UNC119*	-3.5	8.87E-09	137.55	39.53
ENSG00000244198.7	*AC004889.1*	-3.5	3.18E-09	100.99	29.01
ENSG00000122870.12	*BICC1*	-3.5	6.06E-54	211.29	60.59
ENSG00000087253.13	*LPCAT2*	-3.5	2.62E-34	253.01	72.55
ENSG00000204287.14	*HLA-DRA*	-3.5	6.00E-08	72.76	20.82
ENSG00000156510.13	*HKDC1*	-3.5	7.27E-10	84.73	24.22
ENSG00000166741.7	*NNMT*	-3.5	4.17E-19	168.23	48.03
ENSG00000206503.13	*HLA-A*	-3.5	1.81E-83	9178.32	2612.95
ENSG00000227372.12	*TP73-AS1*	-3.5	2.88E-13	397.54	112.96
ENSG00000119778.15	*ATAD2B*	-3.5	2.03E-22	174.74	49.65

ENSG00000135929.9	*CYP27A1*	-3.5	5.56E-16	68.76	19.49
ENSG00000141441.16	*GAREM1*	-3.5	3.35E-07	75.50	21.34
ENSG00000080947.15	*CROCCP3*	-3.5	3.46E-04	43.85	12.36
ENSG00000180758.12	*GPR157*	-3.6	3.03E-25	231.87	65.16
ENSG00000117016.10	*RIMS3*	-3.6	4.03E-04	42.38	11.88
ENSG00000180884.10	*ZNF792*	-3.6	2.06E-34	181.54	50.89
ENSG00000105426.16	*PTPRS*	-3.6	1.12E-37	403.13	112.95
ENSG00000278175.4	*GLIDR*	-3.6	3.71E-04	55.41	15.45
ENSG00000164379.7	*FOXQ1*	-3.6	2.17E-49	748.12	208.06
ENSG00000072954.7	*TMEM38A*	-3.6	2.84E-92	448.12	123.84
ENSG00000178814.17	*OPLAH*	-3.6	4.31E-14	54.56	15.00
ENSG00000152689.18	*RASGRP3*	-3.6	2.92E-06	68.51	18.79
ENSG00000134470.21	*IL15RA*	-3.6	7.89E-95	333.02	91.33
ENSG00000171840.12	*NINJ2*	-3.7	8.49E-12	55.48	15.20
ENSG00000124588.20	*NQO2*	-3.7	2.12E-04	63.30	17.27
ENSG00000164663.14	*USP49*	-3.7	1.10E-07	52.99	14.42
ENSG00000136859.10	*ANGPTL2*	-3.7	4.54E-29	312.92	85.12
ENSG00000148680.16	*HTR7*	-3.7	1.19E-64	501.92	136.11
ENSG00000101079.21	*NDRG3*	-3.7	1.48E-223	1165.10	315.68
ENSG00000183386.10	*FHL3*	-3.7	6.64E-51	322.71	87.13
ENSG00000062524.16	*LTK*	-3.7	1.38E-09	71.52	19.30
ENSG00000265763.4	*ZNF488*	-3.7	1.24E-50	185.00	49.74
ENSG00000143891.17	*GALM*	-3.7	3.30E-35	195.76	52.52
ENSG00000108448.21	*TRIM16L*	-3.7	3.79E-93	465.19	124.74
ENSG00000134775.15	*FHOD3*	-3.7	5.92E-105	646.67	173.11
ENSG00000088881.20	*EBF4*	-3.7	9.15E-06	45.66	12.20
ENSG00000125247.15	*TMTC4*	-3.7	6.22E-03	175.33	46.86
ENSG00000168056.16	*LTBP3*	-3.7	9.13E-05	106.39	28.42
ENSG00000240184.7	*PCDHGC3*	-3.8	1.79E-18	202.35	53.78
ENSG00000071073.13	*MGAT4A*	-3.8	1.25E-09	127.04	33.67
ENSG00000197536.11	*C5orf56*	-3.8	5.71E-13	42.75	11.32
ENSG00000084731.15	*KIF3C*	-3.8	1.29E-38	456.78	120.97
ENSG00000130707.18	*ASS1*	-3.8	4.03E-10	58.67	15.44
ENSG00000115919.15	*KYNU*	-3.8	2.23E-29	115.85	30.46
ENSG00000107738.20	*VSIR*	-3.8	2.17E-11	114.55	30.09
ENSG00000091409.15	*ITGA6*	-3.8	8.17E-54	2635.02	689.98
ENSG00000189120.5	*SP6*	-3.8	1.69E-17	104.47	27.19
ENSG00000181773.7	*GPR3*	-3.9	5.60E-22	112.34	28.77
ENSG00000179588.9	*ZFPM1*	-3.9	4.97E-16	103.95	26.55
ENSG00000119772.16	*DNMT3A*	-3.9	8.25E-17	171.07	43.56
ENSG00000198542.14	*ITGBL1*	-4.0	1.31E-37	147.08	37.21
ENSG00000102996.5	*MMP15*	-4.0	2.04E-19	79.24	19.87
ENSG00000103534.17	*TMC5*	-4.0	4.51E-07	48.05	12.03
ENSG00000141295.14	*SCRN2*	-4.0	4.70E-91	352.44	88.04

ENSG00000078804.13	*TP53INP2*	-4.0	1.53E-94	641.13	160.04
ENSG00000084693.16	*AGBL5*	-4.0	7.12E-79	270.25	67.38
ENSG00000197375.12	*SLC22A5*	-4.0	6.66E-03	85.34	21.24
ENSG00000269609.6	*RPARP-AS1*	-4.0	5.27E-07	58.03	14.44
ENSG00000099282.10	*TSPAN15*	-4.0	8.15E-33	277.27	68.53
ENSG00000157111.13	*TMEM171*	-4.1	9.27E-40	123.95	30.57
ENSG00000204389.10	*HSPA1A*	-4.1	2.94E-134	1334.75	328.77
ENSG00000168994.13	*PXDC1*	-4.1	3.35E-07	51.89	12.72
ENSG00000156711.17	*MAPK13*	-4.1	8.12E-57	1455.30	355.59
ENSG00000150967.18	*ABCB9*	-4.1	1.20E-18	216.90	52.96
ENSG00000163795.14	*ZNF513*	-4.1	8.79E-16	61.39	14.95
ENSG00000142279.13	*WTIP*	-4.1	2.31E-27	529.98	128.58
ENSG00000111801.16	*BTN3A3*	-4.1	1.86E-13	131.41	31.82
ENSG00000187024.14	*PTRH1*	-4.2	3.54E-06	61.53	14.70
ENSG00000172137.19	*CALB2*	-4.2	8.74E-64	646.58	154.06
ENSG00000074410.14	*CA12*	-4.2	1.07E-22	215.69	51.30
ENSG00000099812.9	*MISP*	-4.2	6.85E-41	637.22	150.65
ENSG00000178685.14	*PARP10*	-4.2	5.84E-16	293.92	69.47
ENSG00000197355.11	*UAP1L1*	-4.2	2.82E-69	453.43	107.09
ENSG00000141696.13	*P3H4*	-4.3	4.53E-07	113.01	26.47
ENSG00000283646.2	*LINC02009*	-4.3	1.34E-40	145.29	33.93
ENSG00000204176.14	*SYT15*	-4.3	9.77E-08	70.60	16.44
ENSG00000171310.11	*CHST11*	-4.4	8.82E-62	2556.21	586.86
ENSG00000228594.4	*FNDC10*	-4.4	1.82E-23	86.75	19.78
ENSG00000134508.12	*CABLES1*	-4.4	9.05E-31	231.35	52.04
ENSG00000160469.17	*BRSK1*	-4.4	9.59E-11	74.52	16.76
ENSG00000118707.10	*TGIF2*	-4.5	1.44E-03	73.45	16.50
ENSG00000108846.16	*ABCC3*	-4.5	6.96E-14	278.14	62.48
ENSG00000161682.15	*FAM171A2*	-4.5	1.88E-29	109.27	24.41
ENSG00000142765.18	*SYTL1*	-4.5	5.87E-11	49.81	11.09
ENSG00000172830.13	*SSH3*	-4.5	5.59E-28	173.72	38.60
ENSG00000114779.19	*ABHD14B*	-4.5	5.89E-09	191.78	42.56
ENSG00000126561.16	*STAT5A*	-4.5	6.05E-03	55.55	12.28
ENSG00000162522.11	*KIAA1522*	-4.5	2.65E-73	788.12	174.15
ENSG00000166348.18	*USP54*	-4.5	1.15E-10	242.49	53.54
ENSG00000092621.12	*PHGDH*	-4.6	6.59E-04	93.55	20.49
ENSG00000171408.14	*PDE7B*	-4.6	9.37E-22	75.94	16.61
ENSG00000125967.16	*NECAB3*	-4.6	1.55E-24	261.17	57.08
ENSG00000131435.13	*PDLIM4*	-4.6	1.51E-63	411.73	89.93
ENSG00000010704.18	*HFE*	-4.6	1.08E-17	101.02	22.02
ENSG00000179841.8	*AKAP5*	-4.6	1.41E-20	90.70	19.56
ENSG00000114631.11	*PODXL2*	-4.6	1.88E-24	200.38	43.16
ENSG00000204634.12	*TBC1D8*	-4.7	4.46E-12	114.59	24.51
ENSG00000119630.14	*PGF*	-4.7	7.83E-30	98.28	21.02

ENSG00000069812.11	*HES2*	-4.7	5.35E-06	98.41	21.02
ENSG00000130702.15	*LAMA5*	-4.7	7.84E-09	2900.75	617.75
ENSG00000114993.17	*RTKN*	-4.7	4.55E-34	161.94	34.46
ENSG00000090339.9	*ICAM1*	-4.7	1.34E-14	128.40	27.23
ENSG00000157510.14	*AFAP1L1*	-4.7	4.81E-64	608.30	129.00
ENSG00000104903.5	*LYL1*	-4.7	9.46E-21	56.36	11.91
ENSG00000089486.17	*CDIP1*	-4.7	8.80E-21	473.19	99.72
ENSG00000185112.6	*FAM43A*	-4.8	3.48E-20	302.26	63.56
ENSG00000166033.13	*HTRA1*	-4.8	5.28E-36	803.94	168.94
ENSG00000163803.13	*PLB1*	-4.8	2.14E-13	77.81	16.25
ENSG00000091129.20	*NRCAM*	-4.8	1.25E-05	70.63	14.72
ENSG00000177508.12	*IRX3*	-4.8	8.15E-44	238.32	49.50
ENSG00000187134.14	*AKR1C1*	-4.8	3.78E-14	77.28	16.01
ENSG00000111335.12	*OAS2*	-4.8	2.48E-41	190.58	39.31
ENSG00000151651.16	*ADAM8*	-4.8	1.45E-181	2159.44	445.37
ENSG00000173705.9	*SUSD5*	-4.9	5.75E-44	516.57	106.30
ENSG00000132563.16	*REEP2*	-4.9	2.90E-22	86.84	17.83
ENSG00000006118.14	*TMEM132A*	-4.9	8.87E-40	377.70	77.52
ENSG00000175130.7	*MARCKSL1*	-4.9	0.00E+00	2029.10	414.73
ENSG00000274180.1	*NATD1*	-4.9	4.08E-26	74.97	15.15
ENSG00000124920.13	*MYRF*	-4.9	7.08E-03	43.12	8.71
ENSG00000165912.16	*PACSIN3*	-5.0	1.49E-102	901.44	181.83
ENSG00000088836.14	*SLC4A11*	-5.0	1.11E-42	856.11	171.53
ENSG00000170485.17	*NPAS2*	-5.0	2.82E-40	1280.88	256.39
ENSG00000130720.13	*FIBCD1*	-5.0	5.52E-17	57.69	11.51
ENSG00000143320.9	*CRABP2*	-5.0	2.15E-54	205.96	40.99
ENSG00000185432.12	*METTL7A*	-5.1	5.94E-21	137.29	27.06
ENSG00000135454.14	*B4GALNT1*	-5.1	4.41E-16	70.57	13.88
ENSG00000101417.12	*PXMP4*	-5.1	9.11E-44	205.83	40.33
ENSG00000132182.12	*NUP210*	-5.1	2.39E-06	1420.94	276.41
ENSG00000180448.10	*ARHGAP45*	-5.2	1.41E-30	295.07	57.27
ENSG00000186470.14	*BTN3A2*	-5.2	1.69E-28	229.29	43.90
ENSG00000105655.19	*ISYNA1*	-5.2	7.36E-56	277.10	52.83
ENSG00000170190.16	*SLC16A5*	-5.3	3.47E-28	100.21	19.08
ENSG00000160190.14	*SLC37A1*	-5.3	3.67E-17	80.05	15.24
ENSG00000011332.19	*DPF1*	-5.3	3.88E-07	88.23	16.75
ENSG00000096060.14	*FKBP5*	-5.3	1.42E-04	96.82	18.35
ENSG00000134285.11	*FKBP11*	-5.3	3.83E-58	314.40	59.23
ENSG00000110811.20	*P3H3*	-5.4	2.54E-06	65.26	12.19
ENSG00000166689.16	*PLEKHA7*	-5.4	1.93E-24	537.56	99.94
ENSG00000145040.4	*UCN2*	-5.4	6.73E-31	103.15	19.09
ENSG00000168140.5	*VASN*	-5.4	1.21E-29	87.59	16.14
ENSG00000011028.14	*MRC2*	-5.4	3.73E-227	793.95	146.07
ENSG00000054277.14	*OPN3*	-5.5	5.58E-37	252.17	46.05

ENSG00000173546.7	*CSPG4*	-5.6	1.75E-04	216.47	38.98
ENSG00000115041.13	*KCNIP3*	-5.6	5.51E-20	90.58	16.22
ENSG00000148180.19	*GSN*	-5.6	2.36E-20	296.27	53.01
ENSG00000183914.14	*DNAH2*	-5.6	3.61E-43	275.71	49.12
ENSG00000198756.12	*COLGALT2*	-5.6	2.17E-89	418.35	74.54
ENSG00000169169.14	*CPT1C*	-5.7	3.12E-08	101.47	17.85
ENSG00000176845.13	*METRNL*	-5.7	3.75E-28	94.01	16.36
ENSG00000099337.5	*KCNK6*	-5.8	7.82E-15	150.99	26.26
ENSG00000173801.17	*JUP*	-5.8	1.99E-42	259.22	44.71
ENSG00000136114.17	*THSD1*	-5.8	6.75E-13	44.87	7.71
ENSG00000175315.3	*CST6*	-5.9	3.01E-17	99.65	17.01
ENSG00000232803.1	*SLCO4A1-AS1*	-5.9	1.20E-08	54.01	9.08
ENSG00000123453.18	*SARDH*	-6.0	1.66E-45	147.16	24.60
ENSG00000169515.8	*CCDC8*	-6.0	5.85E-57	159.11	26.39
ENSG00000187764.11	*SEMA4D*	-6.0	1.46E-15	99.09	16.42
ENSG00000108961.14	*RANGRF*	-6.0	2.15E-152	421.38	69.79
ENSG00000181350.12	*LRRC75A*	-6.1	4.62E-33	91.00	15.04
ENSG00000174233.11	*ADCY6*	-6.1	6.84E-08	81.53	13.44
ENSG00000204261.9	*PSMB8-AS1*	-6.1	6.19E-17	47.24	7.76
ENSG00000185513.16	*L3MBTL1*	-6.1	1.07E-13	72.90	11.89
ENSG00000131196.17	*NFATC1*	-6.2	9.32E-23	92.16	14.92
ENSG00000072195.15	*SPEG*	-6.2	2.36E-46	173.36	28.02
ENSG00000198053.11	*SIRPA*	-6.2	5.23E-18	58.16	9.40
ENSG00000143126.8	*CELSR2*	-6.2	4.10E-50	278.31	44.72
ENSG00000197971.15	*MBP*	-6.2	6.66E-106	2160.17	346.54
ENSG00000185499.16	*MUC1*	-6.3	6.57E-18	50.40	8.05
ENSG00000008283.16	*CYB561*	-6.3	6.13E-130	1215.43	193.20
ENSG00000148832.16	*PAOX*	-6.3	2.25E-13	54.23	8.61
ENSG00000170542.6	*SERPINB9*	-6.4	1.07E-103	481.47	75.80
ENSG00000111319.13	*SCNN1A*	-6.4	3.59E-26	103.97	16.19
ENSG00000107882.11	*SUFU*	-6.4	2.24E-19	62.69	9.73
ENSG00000004399.12	*PLXND1*	-6.5	4.52E-07	93.71	14.52
ENSG00000181444.13	*ZNF467*	-6.5	5.38E-16	52.68	8.11
ENSG00000090554.13	*FLT3LG*	-6.5	4.73E-21	53.95	8.25
ENSG00000196917.6	*HCAR1*	-6.6	1.14E-16	50.19	7.58
ENSG00000132688.11	*NES*	-6.7	4.28E-108	448.59	67.16
ENSG00000141524.15	*TMC6*	-6.7	6.06E-13	102.49	15.23
ENSG00000114270.17	*COL7A1*	-6.8	1.71E-08	142.33	20.95
ENSG00000225756.1	*DBH-AS1*	-6.8	5.59E-19	44.12	6.48
ENSG00000088367.23	*EPB41L1*	-6.8	7.35E-54	322.91	47.34
ENSG00000105559.12	*PLEKHA4*	-6.9	2.23E-09	96.65	13.98
ENSG00000232677.8	*LINC00665*	-6.9	5.85E-13	86.21	12.46
ENSG00000170577.8	*SIX2*	-6.9	6.91E-31	135.46	19.56
ENSG00000148175.13	*STOM*	-6.9	1.42E-37	440.16	63.47

ENSG00000153292.16	*ADGRF1*	-6.9	1.09E-10	87.28	12.58
ENSG00000271605.6	*MILR1*	-7.0	1.06E-43	161.14	22.99
ENSG00000107902.14	*LHPP*	-7.1	9.61E-10	47.73	6.74
ENSG00000153404.14	*PLEKHG4B*	-7.2	1.81E-06	50.84	7.08
ENSG00000068079.7	*IFI35*	-7.2	5.59E-28	176.12	24.44
ENSG00000171604.12	*CXXC5*	-7.2	2.46E-40	197.61	27.40
ENSG00000184709.7	*LRRC26*	-7.3	7.97E-24	63.91	8.76
ENSG00000185215.9	*TNFAIP2*	-7.3	4.77E-12	88.05	12.04
ENSG00000173267.14	*SNCG*	-7.3	2.45E-21	62.15	8.46
ENSG00000143416.21	*SELENBP1*	-7.5	1.40E-54	158.78	21.29
ENSG00000161642.17	*ZNF385A*	-7.5	8.22E-13	172.84	23.15
ENSG00000140398.14	*NEIL1*	-7.7	1.09E-08	43.32	5.65
ENSG00000172889.16	*EGFL7*	-7.8	8.36E-108	309.17	39.52
ENSG00000116299.17	*KIAA1324*	-7.9	4.40E-08	45.92	5.85
ENSG00000011105.14	*TSPAN9*	-8.0	1.90E-58	731.09	91.89
ENSG00000088280.19	*ASAP3*	-8.0	1.85E-56	182.22	22.83
ENSG00000232774.8	*AL355916.1*	-8.0	2.73E-43	138.09	17.28
ENSG00000196154.12	*S100A4*	-8.1	6.65E-75	1900.84	234.62
ENSG00000151150.22	*ANK3*	-8.1	8.90E-16	96.58	11.89
ENSG00000073060.16	*SCARB1*	-8.2	2.08E-07	367.94	45.08
ENSG00000182179.13	*UBA7*	-8.2	6.43E-30	111.22	13.56
ENSG00000151388.11	*ADAMTS12*	-8.2	3.88E-39	541.47	65.94
ENSG00000101460.13	*MAP1LC3A*	-8.3	8.67E-22	48.86	5.88
ENSG00000081189.15	*MEF2C*	-8.3	2.30E-15	132.05	15.82
ENSG00000060656.20	*PTPRU*	-8.5	4.28E-21	132.94	15.67
ENSG00000166780.11	*BMERB1*	-8.5	7.70E-23	142.32	16.71
ENSG00000179627.10	*ZBTB42*	-8.5	1.05E-23	89.53	10.49
ENSG00000023892.11	*DEF6*	-8.6	2.59E-45	374.85	43.77
ENSG00000139292.13	*LGR5*	-8.6	2.33E-13	44.01	5.10
ENSG00000133321.11	*PLAAT4*	-8.7	2.18E-34	202.97	23.38
ENSG00000138061.12	*CYP1B1*	-9.0	5.06E-96	1581.87	176.57
ENSG00000204219.11	*TCEA3*	-9.1	1.39E-80	425.97	46.65
ENSG00000184254.17	*ALDH1A3*	-9.1	1.20E-21	127.53	13.96
ENSG00000166165.13	*CKB*	-9.2	8.79E-149	2013.44	219.31
ENSG00000240065.8	*PSMB9*	-9.3	1.08E-37	203.84	21.96
ENSG00000162520.15	*SYNC*	-9.4	9.32E-25	88.69	9.42
ENSG00000198959.12	*TGM2*	-9.5	0.00E+00	5050.71	532.20
ENSG00000254726.3	*MEX3A*	-9.7	8.08E-71	161.79	16.70
ENSG00000177374.13	*HIC1*	-9.7	9.19E-29	90.04	9.26
ENSG00000118898.16	*PPL*	-9.8	4.38E-42	313.58	32.01
ENSG00000148671.14	*ADIRF*	-10.0	1.61E-30	248.26	24.81
ENSG00000239697.11	*TNFSF12*	-10.0	8.35E-05	134.42	13.39
ENSG00000136490.9	*LIMD2*	-10.1	9.18E-60	154.12	15.33
ENSG00000132535.19	*DLG4*	-10.1	2.86E-42	240.16	23.79

ENSG00000189350.12	*TOGARAM2*	-10.1	7.05E-11	41.73	4.12
ENSG00000285294.1	*LINC00842*	-10.1	3.75E-30	67.29	6.63
ENSG00000204282.4	*TNRC6C-AS1*	-10.4	7.65E-09	50.97	4.91
ENSG00000178401.16	*DNAJC22*	-10.4	1.53E-39	106.21	10.19
ENSG00000184916.9	*JAG2*	-10.5	1.29E-36	191.21	18.23
ENSG00000141574.8	*SECTM1*	-10.5	6.39E-59	158.90	15.15
ENSG00000008441.16	*NFIX*	-10.7	2.34E-42	635.88	59.43
ENSG00000152229.18	*PSTPIP2*	-10.7	6.19E-16	49.68	4.64
ENSG00000167600.14	*CYP2S1*	-10.9	8.81E-24	67.16	6.18
ENSG00000132530.17	*XAF1*	-11.2	2.68E-17	42.33	3.79
ENSG00000213366.13	*GSTM2*	-11.3	3.59E-27	58.41	5.18
ENSG00000136848.17	*DAB2IP*	-11.6	1.27E-14	574.08	49.56
ENSG00000197696.10	*NMB*	-11.6	1.49E-21	52.52	4.52
ENSG00000074964.17	*ARHGEF10L*	-11.9	2.18E-17	71.03	5.96
ENSG00000132821.12	*VSTM2L*	-12.3	2.00E-93	491.30	40.08
ENSG00000100473.18	*COCH*	-12.3	2.78E-57	147.40	11.94
ENSG00000156453.13	*PCDH1*	-12.4	1.01E-18	49.76	4.01
ENSG00000197183.14	*NOL4L*	-12.6	4.04E-18	103.83	8.25
ENSG00000204580.13	*DDR1*	-12.6	9.27E-11	303.58	24.05
ENSG00000160867.15	*FGFR4*	-12.7	1.96E-17	52.70	4.15
ENSG00000169220.18	*RGS14*	-12.9	8.27E-08	45.14	3.50
ENSG00000112561.18	*TFEB*	-13.3	1.48E-21	76.22	5.74
ENSG00000110492.15	*MDK*	-13.5	4.71E-48	137.68	10.22
ENSG00000119335.16	*SET*	-13.8	1.14E-26	144.01	10.45
ENSG00000185909.15	*KLHDC8B*	-13.8	5.97E-51	152.10	11.01
ENSG00000117122.14	*MFAP2*	-13.8	3.09E-37	164.87	11.92
ENSG00000101187.16	*SLCO4A1*	-14.4	2.86E-14	380.20	26.49
ENSG00000214435.8	*AS3MT*	-14.4	4.01E-05	101.25	7.05
ENSG00000196739.15	*COL27A1*	-14.4	4.67E-34	243.62	16.92
ENSG00000165376.10	*CLDN2*	-14.6	1.50E-15	45.66	3.12
ENSG00000102934.10	*PLLP*	-14.7	2.13E-14	51.23	3.49
ENSG00000134569.10	*LRP4*	-14.7	7.60E-20	102.41	6.96
ENSG00000264230.9	*ANXA8L1*	-15.1	1.13E-19	62.85	4.17
ENSG00000069122.19	*ADGRF5*	-15.3	8.34E-120	705.35	46.09
ENSG00000083290.20	*ULK2*	-15.5	3.88E-16	188.54	12.20
ENSG00000265190.6	*ANXA8*	-15.7	1.35E-18	54.18	3.46
ENSG00000183421.12	*RIPK4*	-15.8	1.79E-79	199.97	12.62
ENSG00000185436.12	*IFNLR1*	-16.0	4.34E-26	64.01	3.99
ENSG00000198910.14	*L1CAM*	-16.2	2.75E-57	329.77	20.40
ENSG00000182809.10	*CRIP2*	-16.3	2.28E-17	70.52	4.33
ENSG00000119714.11	*GPR68*	-16.4	2.11E-91	246.62	15.06
ENSG00000141068.14	*KSR1*	-16.5	3.19E-40	76.56	4.64
ENSG00000167785.9	*ZNF558*	-16.5	2.14E-08	192.95	11.68
ENSG00000128203.7	*ASPHD2*	-16.9	2.12E-34	79.90	4.73

ENSG00000130881.14	*LRP3*	-17.3	3.97E-36	181.06	10.48
ENSG00000139044.12	*B4GALNT3*	-17.7	3.18E-21	62.90	3.56
ENSG00000116819.8	*TFAP2E*	-18.5	7.79E-31	71.87	3.88
ENSG00000103490.14	*PYCARD*	-19.5	1.11E-30	72.30	3.71
ENSG00000132470.14	*ITGB4*	-20.1	1.16E-42	2168.34	108.06
ENSG00000204228.4	*HSD17B8*	-20.4	2.64E-27	64.22	3.14
ENSG00000105711.12	*SCN1B*	-20.6	3.85E-29	71.98	3.49
ENSG00000163359.15	*COL6A3*	-20.8	7.03E-51	173.18	8.34
ENSG00000095752.7	*IL11*	-20.9	2.41E-34	1808.74	86.61
ENSG00000110446.11	*SLC15A3*	-22.9	3.68E-13	42.78	1.87
ENSG00000121753.12	*ADGRB2*	-25.3	6.14E-17	45.05	1.78
ENSG00000143845.15	*ETNK2*	-26.3	1.47E-42	140.55	5.33
ENSG00000182752.10	*PAPPA*	-26.4	1.69E-65	215.77	8.18
ENSG00000182272.12	*B4GALNT4*	-26.4	6.37E-18	61.07	2.31
ENSG00000100979.15	*PLTP*	-28.0	5.33E-25	64.15	2.29
ENSG00000146197.9	*SCUBE3*	-28.6	4.95E-19	44.95	1.57
ENSG00000105619.13	*TFPT*	-29.7	1.99E-24	68.17	2.30
ENSG00000166897.15	*ELFN2*	-30.4	1.58E-114	375.74	12.35
ENSG00000106538.10	*RARRES2*	-31.1	1.40E-24	72.62	2.34
ENSG00000115468.12	*EFHD1*	-31.4	1.76E-113	303.69	9.68
ENSG00000106484.16	*MEST*	-32.9	1.33E-79	322.97	9.80
ENSG00000198246.9	*SLC29A3*	-34.9	1.59E-52	132.04	3.79
ENSG00000137486.17	*ARRB1*	-35.4	3.73E-39	242.58	6.86
ENSG00000151715.8	*TMEM45B*	-35.5	3.18E-16	40.75	1.15
ENSG00000197122.11	*SRC*	-35.5	3.44E-27	168.85	4.76
ENSG00000127324.9	*TSPAN8*	-35.7	4.17E-137	388.58	10.89
ENSG00000144152.13	*FBLN7*	-37.1	8.57E-21	59.36	1.60
ENSG00000165949.12	*IFI27*	-37.1	2.00E-13	74.35	2.00
ENSG00000179083.6	*FAM133A*	-38.2	5.30E-24	60.39	1.58
ENSG00000115226.10	*FNDC4*	-39.0	2.31E-18	47.55	1.22
ENSG00000196172.9	*ZNF681*	-40.8	3.37E-24	75.18	1.84
ENSG00000126878.13	*AIF1L*	-42.5	1.65E-49	147.29	3.46
ENSG00000166831.9	*RBPMS2*	-43.3	4.36E-20	55.62	1.29
ENSG00000185585.20	*OLFML2A*	-43.9	4.44E-47	249.54	5.69
ENSG00000146966.13	*DENND2A*	-44.7	7.87E-16	47.21	1.06
ENSG00000142235.10	*LMTK3*	-45.2	2.35E-22	66.73	1.48
ENSG00000128271.22	*ADORA2A*	-45.7	1.97E-09	43.36	0.95
ENSG00000163362.11	*INAVA*	-47.8	1.34E-21	66.84	1.40
ENSG00000104870.13	*FCGRT*	-48.1	2.87E-71	210.46	4.37
ENSG00000131746.13	*TNS4*	-48.3	4.24E-58	404.65	8.38
ENSG00000131095.13	*GFAP*	-51.1	4.37E-21	55.11	1.08
ENSG00000142173.15	*COL6A2*	-53.5	9.82E-25	87.18	1.63
ENSG00000142347.19	*MYO1F*	-55.4	1.65E-22	70.20	1.27
ENSG00000198768.11	*APCDD1L*	-60.8	3.56E-75	253.71	4.17

ENSG00000103888.17	*CEMIP*	-61.2	1.65E-48	684.52	11.19
ENSG00000131459.13	*GFPT2*	-61.7	8.42E-175	1004.92	16.29
ENSG00000173432.12	*SAA1*	-61.8	1.88E-21	73.58	1.19
ENSG00000125170.11	*DOK4*	-76.9	2.12E-12	69.41	0.90
ENSG00000185885.16	*IFITM1*	-86.9	4.15E-16	60.58	0.70
ENSG00000135373.12	*EHF*	-88.9	2.38E-18	49.44	0.56
ENSG00000268119.6	*AC010615.2*	-89.9	4.33E-17	61.26	0.68
ENSG00000078900.15	*TP73*	-94.0	4.02E-16	64.77	0.69
ENSG00000180616.9	*SSTR2*	-98.1	1.64E-14	51.42	0.52
ENSG00000114812.13	*VIPR1*	-100.8	8.16E-20	84.24	0.84
ENSG00000276850.5	*AC245041.2*	-107.8	1.80E-36	137.60	1.28
ENSG00000183876.9	*ARSI*	-108.5	1.03E-14	55.71	0.51
ENSG00000211772.11	*TRBC2*	-109.1	7.40E-14	40.99	0.38
ENSG00000157303.11	*SUSD3*	-112.1	1.50E-19	83.23	0.74
ENSG00000106976.20	*DNM1*	-113.9	6.82E-38	184.56	1.62
ENSG00000177679.16	*SRRM3*	-126.0	3.13E-16	66.60	0.53
ENSG00000170369.4	*CST2*	-154.1	2.04E-15	41.45	0.27
ENSG00000184985.16	*SORCS2*	-156.8	1.05E-18	98.78	0.63
ENSG00000116132.12	*PRRX1*	-186.7	5.12E-16	47.26	0.25
ENSG00000112655.16	*PTK7*	-192.5	6.77E-58	488.10	2.54
ENSG00000206195.11	*DUXAP8*	-213.8	8.27E-17	41.57	0.19
ENSG00000151692.15	*RNF144A*	-218.5	1.57E-15	42.11	0.19
ENSG00000170801.10	*HTRA3*	-221.3	9.90E-19	59.01	0.27
ENSG00000099864.18	*PALM*	-229.2	6.04E-19	63.73	0.28
ENSG00000215845.11	*TSTD1*	-233.1	2.72E-19	44.92	0.19
ENSG00000085117.12	*CD82*	-234.2	1.77E-112	915.93	3.91
ENSG00000214049.8	*UCA1*	-241.4	9.49E-20	106.01	0.44
ENSG00000187922.14	*LCN10*	-242.1	2.12E-18	46.76	0.19
ENSG00000101210.12	*EEF1A2*	-253.5	3.47E-195	1344.54	5.30
ENSG00000130635.15	*COL5A1*	-259.1	1.04E-22	61.02	0.24
ENSG00000113504.21	*SLC12A7*	-259.9	1.41E-57	440.41	1.69
ENSG00000197249.13	*SERPINA1*	-285.2	1.81E-18	55.05	0.19
ENSG00000244306.11	*DUXAP10*	-347.2	2.08E-20	67.32	0.19
ENSG00000152128.13	*TMEM163*	-374.1	2.16E-30	263.92	0.71
ENSG00000028137.19	*TNFRSF1B*	-466.4	8.55E-24	125.53	0.27
ENSG00000170412.18	*GPRC5C*	-582.0	4.42E-26	112.48	0.19
ENSG00000105894.12	*PTN*	-604.4	1.18E-26	116.75	0.19
ENSG00000163083.6	*INHBB*	-650.0	5.54E-29	341.92	0.53
ENSG00000240225.10	*ZNF542P*	-761.4	1.23E-28	147.27	0.19
ENSG00000101017.14	*CD40*	-1257.6	2.08E-33	340.89	0.27
ENSG00000130303.13	*BST2*	-1287.8	2.05E-32	405.59	0.31
ENSG00000162458.13	*FBLIM1*	-1479.7	1.22E-34	286.39	0.19
ENSG00000188636.4	*RTL6*	-2238.7	2.57E-39	843.11	0.38
ENSG00000162511.8	*LAPTM5*	-4941.9	2.01E-47	1334.06	0.27
